# Application of multimodal deep learning and multi-instance learning fusion techniques in predicting STN-DBS outcomes for Parkinson's disease patients

**DOI:** 10.1016/j.neurot.2024.e00471

**Published:** 2024-10-16

**Authors:** Bowen Chang, Zhi Geng, Jiaming Mei, Zhengyu Wang, Peng Chen, Yuge Jiang, Chaoshi Niu

**Affiliations:** a Department of Neurosurgery, The First Affiliated Hospital of USTC, Division of Life Sciences and Medicine, University of Science and Technology of China, Hefei, Anhui Province, PR China; b Anhui Province Key Laboratory of Brain Function and Brain Disease, Hefei, Anhui Province, PR China; c Department of Neurology, The First Affiliated Hospital of Anhui Medical University, Hefei, Anhui Province, PR China; d Key Laboratory of Xin'an Medicine, Ministry of Education, Anhui Province Key Laboratory of R&D of Chinese Medicine, Anhui University of Chinese Medicine, Hefei, Anhui Province, PR China

**Keywords:** Parkinson's disease, Deep brain stimulation, Deep learning, Multi-instance, Prediction model

## Abstract

Parkinson's Disease (PD) is a progressive neurodegenerative disorder with substantial impact on patients' quality of life. Subthalamic nucleus deep brain stimulation (STN-DBS) is an effective treatment for advanced PD, but patient responses vary, necessitating predictive models for personalized care. Recent advancements in medical imaging and machine learning offer opportunities to enhance predictive accuracy, particularly through deep learning and multi-instance learning (MIL) techniques. This retrospective study included 127 PD patients undergoing STN-DBS. Medical records and imaging data were collected, and patients were categorized based on treatment outcomes. Advanced segmentation models were trained for automated region of interest (ROI) delineation. A novel 2.5D deep learning approach incorporating multi-slice representation was developed to extract detailed ROI features. Multi-instance learning fusion techniques integrated predictions across multiple slices, combining radiomics and deep learning features to enhance model performance. Various machine learning algorithms were evaluated, and model robustness was assessed using cross-validation and hyperparameter optimization. The MIL model achieved an area under the curve (AUC) of 0.846 for predicting STN-DBS outcomes, surpassing the radiomics model's AUC of 0.825. Integration of MIL and radiomics features in the DLRad model further improved discriminative ability to an AUC of 0.871. Calibration tests showed good model reliability, and decision curve analysis demonstrated clinical utility, affirming the model's predictive advantage. This study demonstrates the efficacy of integrating MIL, radiomics, and deep learning techniques to predict STN-DBS outcomes in PD patients. The multimodal fusion approach enhances predictive accuracy, supporting personalized treatment planning and advancing patient care.

## Introduction

Parkinson's Disease (PD) is a progressive neurodegenerative disorder characterized by motor and non-motor symptoms that severely impact patients' quality of life [1]. Subthalamic nucleus deep brain stimulation (STN-DBS) is an effective treatment for advanced PD, proven to alleviate motor symptoms and improve functional outcomes [[2], [3], [4]]. However, patient responses to STN-DBS vary significantly, highlighting the need for robust and convenient predictive models to screen suitable patients and optimize care [5,6]. Recent advancements in medical imaging and machine learning have opened new avenues for improving the predictive accuracy of clinical outcomes for PD patients undergoing STN-DBS [7]. For instance, studies by Boutet et al. demonstrated that functional magnetic resonance imaging (fMRI) combined with machine learning can predict optimal stimulation parameters for DBS, while Chen et al. successfully improved predictive accuracy by employing a machine learning model based on functional connectivity [8]. Together, these works indicate that integrating advanced machine learning and medical imaging technologies can effectively enhance the clinical outcomes for patients undergoing STN-DBS [9].

Traditional radiomics and connectomics approaches rely on handcrafted features extracted from medical images and have shown potential in offering predictive insights [10,11]. However, these models often fail to capture the complex, multi-dimensional nature of medical data. In contrast, deep learning models have demonstrated promising results due to their ability to automatically learn and extract complex features from large datasets.

In previous studies, most imaging models predicting DBS efficacy have been based on functional connectivity. However, fMRI imaging data require complex preprocessing and analysis, as well as relatively high demands for scanning quality and time. In this study, we attempt to construct a model using structural brain images from patients. Subtle structural changes observed in T1-weighted MRI images may impact functional outcomes of neurological disorders such as PD by influencing neural pathways critical to disease progression and response to DBS. This hypothesis is supported by a series of studies that have demonstrated associations between changes in brain structure, such as variations in gray matter volume, and functional outcomes. The work conducted by Fitzhugh et al. explored how longitudinal changes in resting-state functional connectivity and gray matter volume are associated with the conversion to hearing impairment in older adults [12]. This study suggests that structural changes have a significant impact on functional capacities, hinting at broader applicability of this concept that could extend to PD and DBS outcomes. Similarly, the research by Qian et al. adds weight to this argument by showing that brain gray matter volume and functional connectivity are directly correlated with outcomes in smoking cessation [13]. This evidence underscores the intertwined nature of structural and functional brain changes, reinforcing the idea that structural alterations measurable through T1-weighted MRI may underlie significant functional shifts. In a more direct investigation into PD, a study comparing clinical outcomes and connectivity in awake versus asleep deep brain stimulation found that clinical outcomes and electrode placement, as indirect measures of DBS-induced structural changes, can achieve optimal targeting based on connectivity estimates, thereby influencing functional outcomes [14]. The aforementioned studies reveal the predictive value of brain structural changes, particularly those discernible through T1-weighted MRI, in functional outcomes across various conditions, including PD. This insight not only substantiates the hypothesis driving the current study but also lays a foundation for future research aimed at refining the targeting and efficacy of interventions like STN-DBS in PD by leveraging detailed insights into brain structure-function relationships.

**Fig. 1:**
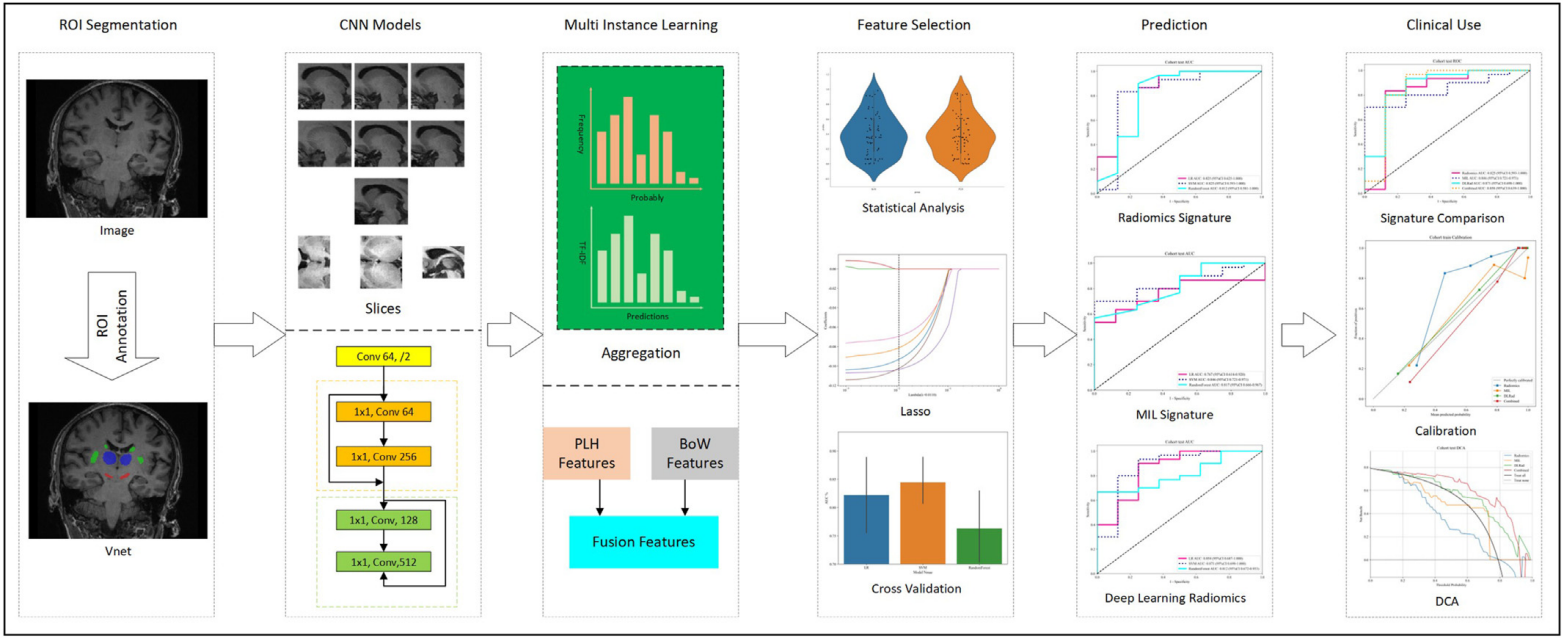
Workflow of this work. Image Acquisition: Acquiring T1-weighted MRI scans from patients; ROI Segmentation: Identifying and delineating brain structures crucial for predicting STN-DBS outcomes, such as the substantia nigra, striatum, and thalamus; Slice-Level Model Training: Training a 2.5D deep learning model using segmented ROIs from multiple adjacent slices to capture 3D information; Multi-instance Learning Fusion: Aggregating predictions from multiple slices within the ROI using multi-instance learning techniques, such as Prediction Likelihood Histogram (PLH) and Bag of Words (BoW), and further combining with radiomics features; Feature Selection: Refining the feature set by removing redundant information through dimensionality reduction techniques like t-tests, correlation coefficients, and Lasso regularization; Prediction: Building a final prediction model using machine learning algorithms like logistic regression, support vector machines, and random forests.

This study aims to develop a comprehensive predictive model by integrating multi-instance learning (MIL) techniques with radiomics and deep learning approaches. By employing a 2.5D data representation and advanced segmentation models, we seek to enhance the predictive power for STN-DBS outcomes in PD patients. Additionally, our research investigates the benefits of incorporating 2.5D data processing can enhance the accuracy of regional features within a single imaging modality, although these methods have primarily focused on T1-weighted imaging (Fig. 1).

We retrospectively collected medical records and imaging data from PD patients who underwent STN-DBS treatment. This study rigorously evaluates various machine learning algorithms and their performance in predicting clinical outcomes, focusing on model calibration, discriminative ability, and potential clinical applications. The primary goal of this research is to improve the precision and reliability of outcome predictions for PD patients undergoing STN-DBS, thereby facilitating better individualized treatment planning and enhancing overall patient care.

## Methods

### Patients

Medical records and questionnaire results were retrospectively collected from 127 PD patients who underwent STN-DBS treatment at the First Hospital of University of Science and Technology of China between September 2018 and April 2022. The study protocol was approved by the hospital's ethics committee (2022-RE-154). Inclusion criteria: 1) Age ≥18 years; 2) Meets the diagnostic criteria of the UK Parkinson's Disease Society and diagnosed with Parkinson's Disease by a senior neurosurgeon. Exclusion criteria: 1) Secondary brain lesions due to trauma, cerebral infarction, cerebral hemorrhage, cerebrovascular malformation, brain tumors, etc.; 2) Patients who had undergone previous brain surgery (including interventional and open surgeries); 3) Patients who were lost to follow-up after DBS surgery; 4) Patients who experienced significant disease progression within two years or received other interventions.

Demographic and clinical variables, including age, gender, disease duration, and Hoehn-Yahr (H–Y) stage, were collected from patient records and questionnaires. Symptom severity was assessed using the Unified Parkinson's Disease Rating Scale Part III (UPDRS-III) in both medicated and unmedicated states (Med-on/off). Patients' motor symptoms were re-evaluated using the UPDRS-III scale two years postoperatively in the post med off/stim on state, and the UPDRS-III improvement rate was calculated as follows: ([Preop Med-off - Postop Med-off]/Preop Med-off). During the med-off assessment, patients underwent a withdrawal period of 12 ​h, during which all anti-Parkinson medications were discontinued. The med-on assessment was conducted within 1–1.5 ​h after patients took their usual dosage of anti-Parkinson medications to ensure that the drugs had reached their optimal effect. We selected the UPDRS score evaluated at 2 years post-surgery as a key follow-up measure. This time point is generally considered a reasonable window for analyzing the efficacy of STN-DBS [15,16]. By this stage, patients have typically experienced significant improvement post-surgery, allowing for a better assessment of the impact of surgical treatment on their symptoms. While we acknowledge that disease progression can influence UPDRS scores, to minimize this confounding factor, we implemented stringent inclusion criteria ensuring that participants exhibited relatively stable disease progression both before and after surgery. The severity of symptoms in enrolled patients was not significant when the UPDRS-III Med-off score was assessed with DBS stimulation off at 2 years (Table 1). This provides a more reliable basis for evaluating surgical outcomes. Patients were categorized into either the relief group (the improvement rate was greater than 0) or the non-relief group (The improvement rate is less than or equal to 0) based on their UPDRS-III improvement rate. This study divided the samples into two cohorts: a training cohort, comprising 70 ​% of the data, and an internal validation cohort, consisting of the remaining 30 ​%.

**Table 1:** Baseline characters of our cohorts.

Feature name	All	train	test	*P*-value
No.	127	89	38	
Age	59.63 ​± ​7.35	59.69 ​± ​6.93	59.50 ​± ​8.36	0.897
Gender				0.545
Male	57 (44.88)	42 (47.19)	15 (39.47)	
Female	70 (55.12)	47 (52.81)	23 (60.53)	
UPDRS-III Med-on Preop	54.15 ​± ​12.06	54.27 ​± ​9.26	54.03 ​± ​13.31	0.765
UPDRS-III Med-off Preop	59.63 ​± ​7.35	58.75 ​± ​6.23	59.55 ​± ​8.15	0.432
UPDRS-III Med-off Postop (stimulation off)	61.26 ​± ​12.06	60.83 ​± ​13.23	61.38 ​± ​10.36	0.526
UPDRS-III Med-off Postop (stimulation on)	25.06 ​± ​9.81	25.26 ​± ​4.03	25.12 ​± ​8.15	0.332
The improvement rate of levodopa	0.57 ​± ​0.13	0.57 ​± ​0.08	0.57 ​± ​0.18	0.667
DBS improvement rate	0.51 ​± ​0.29	0.52 ​± ​0.11	0.51 ​± ​0.09	0.346
Duration	9.36 ​± ​4.41	9.37 ​± ​2.28	9.36 ​± ​1.35	0.562
Outcome				0.733
Relief	99 (77.95)	69 (77.52)	30 (78.94)	
Non-relief	28 (22.05)	20 (22.48)	8 (21.06)	
H–Y staging				0.862
2.5	22 (17.32)	16 (17.98)	6 (15.79)	
3	65 (51.18)	45 (50.56)	20 (52.63)	
4	40 (31.50)	28 (31.46)	12 (31.58)	

UPDRS-III: Unified Parkinson's Disease Rating Scale Part III; H–Y: Hoehn-Yahr; DBS: Deep Brain Stimulation.

### Image acquisition

MRI images for all patients were acquired three days before the STN-DBS treatment to ensure the timeliness and relevance of the imaging data. For all subjects, T1-weighted MRI were performed using a 3.0 ​T MRI scanner (Discovery MR750; GE Healthcare, Chicago, IL, USA) with an eight-channel phased-array head coil. Earplugs were placed in the subjects' ears prior to scanning to isolate noise. Participants were then asked to use foam pads to stabilize their heads in order to minimize involuntary movements. Structural images were acquired using a sagittal magnetization prepared rapid gradient echo three dimensional T1-weighted sequence [repetition time (TR) ​= ​8.5 ​ms, echo time (TE) ​= ​3.2 ​ms, inversion time (TI) ​= ​450 ​ms, and flip angle (FA) ​= ​12°.

### Image segmentation

In our experiment, we first used ITK-SNAP to delineate Regions of Interest (ROIs) in the training set. In this study, we selected the substantia nigra (SN), striatum, and thalamus as regions of interest (ROI) due to their significant roles in the pathophysiology of Parkinson's disease and the existing literature supporting their association with improvements in motor function [17]. While acknowledging the importance of the STN as the primary DBS target, constraints related to imaging resolution limited our ability to reliably analyze this small-region structure across all patients. Our approach was guided by a hypothesis-driven selection of regions where structural changes were expected to be most apparent and detectable given the imaging capabilities and prior research findings. We recognize the need for future research to incorporate a wider array of motor-related regions, including the STN, as advancements in imaging techniques evolve. The delineation was carried out sequentially by two experienced neurosurgeons. The initial manual delineation was subsequently reviewed by a senior neurosurgeon with over 20 years of experience. This initial manual delineation served as the training data for developing an automated segmentation model.

**Fig. 2:**
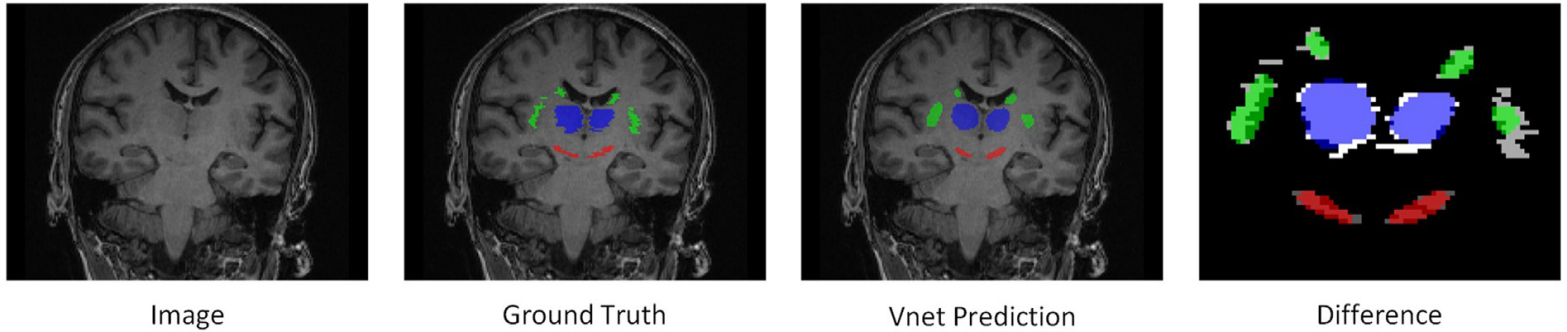
Visualization of ROI segmentation Results. The above illustration presents the recognition results of our V-Net. In the rightmost “Diff” section, it can be observed that the differences in recognition accuracy are minimal. Such discrepancies are considered negligible within our deep learning workflow. This effectively validates the feasibility of the automatic delineation process we have proposed.

We then trained three advanced segmentation models—Segres-net, U-net, and V-net—specifically designed for automated ROI segmentation. The purpose of utilizing these models was to minimize the need for manual delineation, thereby enhancing the efficiency and reproducibility of ROI identification in medical imaging. The trained models were then applied to a separate test dataset to automatically predict and delineate all ROIs (Fig. 2).

### D multi-instance learning

While 3D T1 images provide comprehensive structural information, training 3D convolutional networks poses significant complexity and computational demands. Moreover, 3D models necessitate larger datasets to mitigate the risk of overfitting, a challenge given our relatively limited data volume. To address these issues, we implemented a 2.5D approach that employs a multi-view and multi-slice strategy, effectively reducing model complexity while preserving adequate 3D information for precise predictions. This method is particularly advantageous under computational constraints. In the rapidly advancing field of deep learning, traditional models often rely on the maximum cross-section of the region of interest (ROI), which can result in the neglect of crucial contextual information within the ROI. To counter this limitation, we have integrated the 3D characteristics of the ROI into our model design. Our 2.5D deep learning model enhances feature representation by incorporating several adjacent slices to the central slice and merging data from multiple angles, allowing for a more nuanced and detailed description of the ROI.

### Data generation

In our data generation process, we extracted multiple slices from T1-weighted MRI images. To fully capture the structural information of the ROI, we utilized slices at positions 0, ±1, ±2, and ±4 (yielding a total of seven regions, with 0 representing the maximum ROI cross-section) and acquired images from three different orientations (axial, coronal, and sagittal, resulting in three additional regions). This approach produced a total of ten distinct two-dimensional regions. Such diversity enables the model to learn features from various perspectives. We employed the OKT-crop_max_roi tool from the OnekeyAI platform for cropping, with parameters set to include slices at positions +1, +2, −1, −2, +4, and −4.

### Slice-level model training

During the model training phase, we employed the 2.5D data generated within the deep learning framework to assess its effectiveness. We evaluated the performance of several advanced deep learning architectures, specifically DenseNet121, ResNet101, and InceptionV3, aiming to enhance the capabilities of traditional convolutional neural networks (CNNs). Through comparative analyses, we focused on specific performance metrics to identify the model that best aligns with our research objectives.

To standardize the intensity distribution across RGB channels, we applied Z-score normalization. During training, we utilized real-time data augmentation techniques, including random cropping and horizontal and vertical flipping, while only normalization was applied to test images. Additionally, we standardized the grayscale values of the slices using min-max transformation and resized the images to 224 ​× ​224 pixels (or 299 ​× ​299 pixels for InceptionV3) using nearest neighbor interpolation.

To enhance model generalization, we implemented a learning rate adjustment strategy based on cosine decay, defined by the equation:

$$\eta_{t}=\eta_{\min}^{i}+\frac{1}{2}\left(\eta_{\max}^{i}-\eta_{\min}^{i}\right)\left(1+\cos\left(\frac{T_{cur}}{T_{i}}\pi \right)\right)$$

where $\eta_{\min}^{i}=0$ and $\eta_{\max}^{i}=0.01$. Stochastic Gradient Descent (SGD) was utilized as the optimizer, and softmax cross-entropy was employed as the loss function.

### Multi-instance learning fusion

In our study, we implemented two multi-instance learning fusion techniques. Using the 2.5D deep learning model, we created a Prediction Likelihood Histogram (PLH) that lists the predicted probabilities and labels for each slice, providing a probabilistic summary of prediction outcomes. Employing the Bag of Words (BoW) method, we sliced and extracted data from each image, resulting in seven predictions per sample, which were analyzed using the Term Frequency-Inverse Document Frequency (TF-IDF) method [18,19]. We enhanced our model by integrating PLH and BoW features with radiomics data, utilizing different data sources to improve the representation capability and accuracy of our classification task.

### Signature building

For the aggregated multi-instance learning features, we employed dimensionality reduction techniques to refine our feature set. To optimize our feature set, we initially assessed feature importance using t-tests, removing non-significant features with p-values greater than 0.05. Next, we employed Pearson correlation coefficients to identify highly correlated feature pairs (correlation coefficient >0.9) and excluded one of each pair to reduce collinearity. Finally, we applied LASSO regularization within a 10-fold cross-validation framework for further feature reduction, ensuring the robustness and effectiveness of the selected feature combination. These features were modeled using popular machine learning algorithms, including logistic regression, support vector machines, and random forests. To address the issue of sample imbalance, we applied the SMOTE method during the training process. To ensure the robustness of the model, we utilized 5-fold cross-validation in the training dataset and optimized hyperparameters through grid search. Additionally, to evaluate the effectiveness of our multi-instance learning approach, we compared three different aggregation methods: maximum, minimum, and average values.

We evaluated the predictive performance of classical radiomics models using handcrafted features. We benchmarked this traditional approach against our deep learning model to highlight the strengths and limitations of each. The comprehensive radiomics modeling process and outcomes are detailed in Supplementary Material 3A. We performed feature-level fusion of selected 2.5D deep learning and radiomics features and modeled them using machine learning algorithms such as logistic regression, SVM, and random forests. This study aims to assess the ability of the fusion model to identify the target. The diagnostic performance of the deep learning model in the test cohort was evaluated by constructing Receiver Operating Characteristic (ROC) curves. The DeLong test, applied to both training and testing sets. Calibration performance was assessed using calibration curves and the Hosmer-Lemeshow goodness-of-fit test was used to evaluate its reliability. Additionally, clinical utility of the predictive model was determined through Decision Curve Analysis (DCA).

### Statistical analysis

We used the Shapiro-Wilk test to evaluate the normality of clinical characteristics. Continuous variables were assessed for significance using either the *t*-test or the Mann-Whitney *U* test, depending on their distribution. Categorical variables were analyzed using the Chi-square (χ^2^) test. Baseline characteristics for all cohorts are presented in Table 1. The p-values between different cohorts were all greater than 0.05, indicating no significant differences and confirming no biased partitioning between groups.

Data analysis was conducted using Python 3.7.12 on the OnekeyAI platform version 3.5.12. Statistical analysis was performed with statsmodels version 0.13.2. Radiomics feature extraction was carried out using PyRadiomics version 3.0.1. Machine learning algorithms, including support vector machines (SVM), were implemented using Scikit-learn version 1.0.2. Our deep learning model was developed with PyTorch version 1.11.0 and performance optimization was done using CUDA version 11.3.1 and cuDNN version 8.2.1.

## Results

### Clinical features

This study ultimately included a total of 127 PD patients, who were divided into a training cohort of 89 patients and a test cohort of 38 patients in a 7:3 ratio. There were no significant differences in demographic data and clinical scores between the training and test cohorts (Table 1). In our study, we conducted comprehensive univariate analyses on all clinical features, focusing on calculating the odds ratio (OR) and the corresponding *P*-value for each variable. Age was specifically used in the construction of the final fusion model (Table 2).

**Table 2:** Univariable Analysis of clinical features.

Feature name	OR	OR lower 95%CI	OR upper 95%CI	*P*-value
Age	0.987	0.977	0.998	0.017
Gender	1.026	0.883	1.190	0.778
UPDRS-III Med-on Preop	1.086	0.981	1.143	0.386
UPDRS-III Med-off Preop	1.069	0.836	1.127	0.229
The improvement rate of levodopa	1.025	0.975	1.083	0.218
Duration	1.127	0.998	1.258	0.115
H–Y staging	1.182	0.876	1.248	0.943

UPDRS-III: Unified Parkinson's Disease Rating Scale Part III; H–Y: Hoehn-Yahr; OR: Odds Ratio; CI: Confidence Interval.

### Signature comparison

**Fig. 3:**
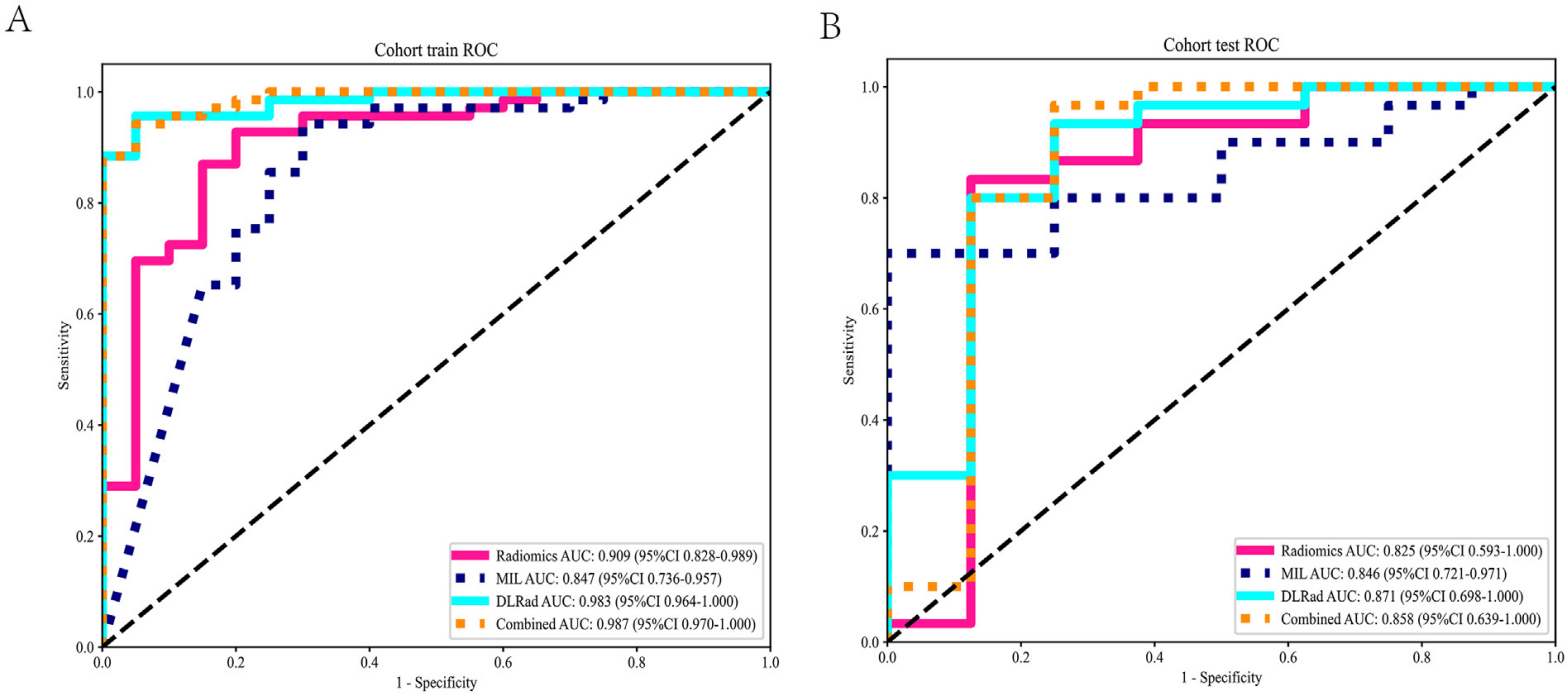
Different signatures' ROC on different cohort. A. training cohort; B. test cohort.

Table 3 showed that in the test cohort, the AUC of the MIL model was 0.846, outperforming the radiomics model's AUC of 0.825. This suggests that MIL features may better capture the nuances necessary for effective prediction in this context. The DLRad model, which integrates MIL and radiomics features, further improved discriminative ability, achieving an AUC of 0.871 (Fig. 3). This enhancement indicates that combining these different data modalities can produce a more robust and generalizable model. However, when Age was added to create the Combined model, the AUC slightly decreased to 0.858, suggesting that Age did not add predictive value to the model and may potentially dilute the effectiveness of the main predictive features. For more details on the results of the MIL, Radiomics, and DLRad models, see Supplementary 2A, 2B, 3A, and 4A. We created confusion matrices for both the training and testing phases, as shown in Supplementary 5A, to evaluate the specific classification performance of the model. They help us identify which sample categories are more prone to misclassification and understand the classification biases of the model.

**Table 3:** Metrics on different signature.

Signature	Accuracy	AUC	95 ​% CI	Sensitivity	Specificity	PPV	NPV	Cohort
Radiomics	0.888	0.909	0.8284–0.9890	0.913	0.800	0.940	0.727	Train
MIL	0.876	0.847	0.7362–0.9573	0.928	0.700	0.914	0.737	Train
DLRad	0.944	0.983	0.9636–1.0000	0.942	0.950	0.985	0.826	Train
Combined	0.933	0.986	0.9676–1.0000	0.928	0.950	0.985	0.792	Train
Radiomics	0.474	0.825	0.5935–1.0000	0.367	0.875	0.917	0.269	Test
MIL	0.684	0.846	0.7210–0.9707	0.600	1.000	1.000	0.400	Test
DLRad	0.789	0.871	0.6978–1.0000	0.800	0.750	0.923	0.500	Test
Combined	0.891	0.858	0.6478–1.0000	1.000	0.625	0.909	1.000	Test

MIL: Multi-instance Learning; DLRad: Deep Learning Radiomics; AUC: Area Under Curve; CI: Confidence Interval; PPV: Positive Predictive Value; NPV: Negative Predictive Value.

**Fig. 4:**
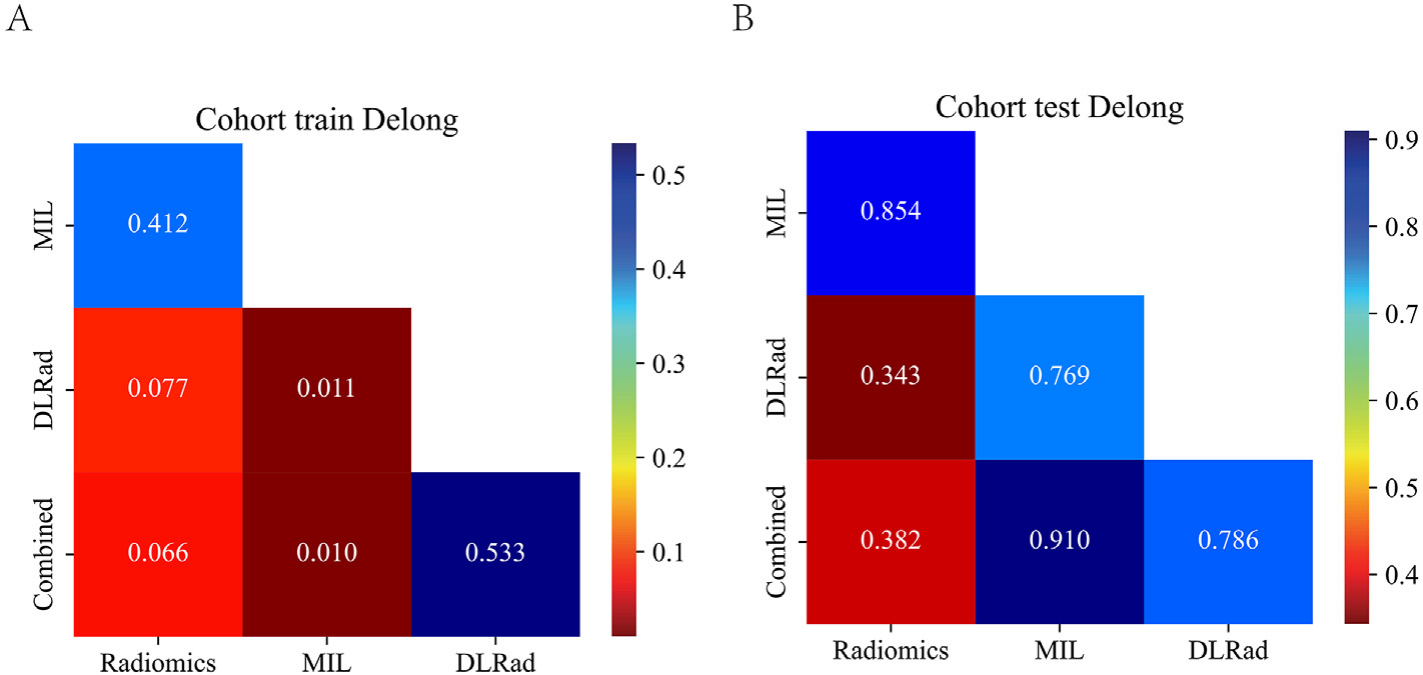
DeLong test results for different signatures. A. training cohort; B. test cohort.

In the DLRad model, the integration of MIL and radiomics features significantly enhanced model performance, with the highest observed AUC in the tested configurations confirming this. The decrease in AUC with the addition of Age in the Combined model highlights the importance of feature selection in model development, not all available data contributes positively to model accuracy. This finding underscores the necessity of selectively incorporating features that truly enhance predictive capability, especially in complex models aimed at disease prediction. Fig. 4 showed that in the training cohort, the combined signature achieved the highest AUC, followed by DLRad, MIL, and radiomics, respectively. The DeLong test result indicates that the combined signature performs significantly better than radiomics and DLRad. In the test cohort, the DLRad signature achieved the highest AUC, followed by combined, MIL, and radiomics, respectively. The DeLong test result indicates that the DLRad signature performs significantly better than radiomics, MIL and combined. The study emphasizes the effectiveness of multimodal feature fusion over single-source data, advocating for the strategic integration of various data types to improve the generalizability and reliability of medical diagnostic prediction models.

**Fig. 5:**
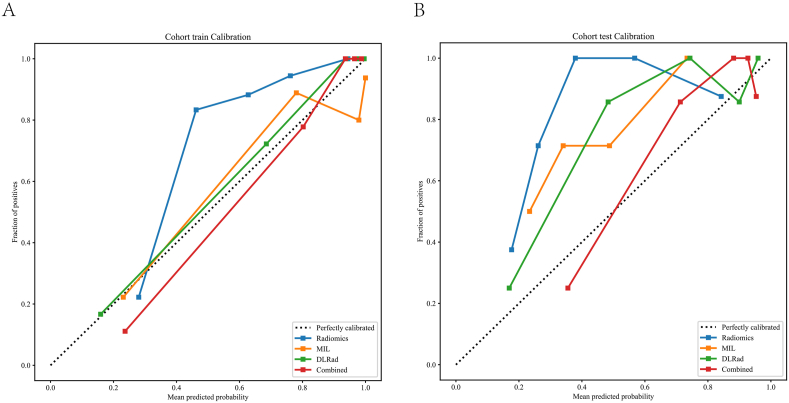
Calibration curves of different signatures for the test cohort. A. training cohort; B. test cohort.

The Hosmer-Lemeshow (HL) test was used to quantify the discrepancy between predicted probabilities and observed outcomes, with lower HL statistics indicating better model calibration, meaning the model's predictions are closer to actual outcomes. In this study, the Combined model demonstrated superior calibration performance. The HL test statistic for the training set was 0.262, and for the test set was 0.147, with p-values >0.05, indicating good calibration for both datasets (Fig. 5).

### Clinical use

**Fig. 6:**
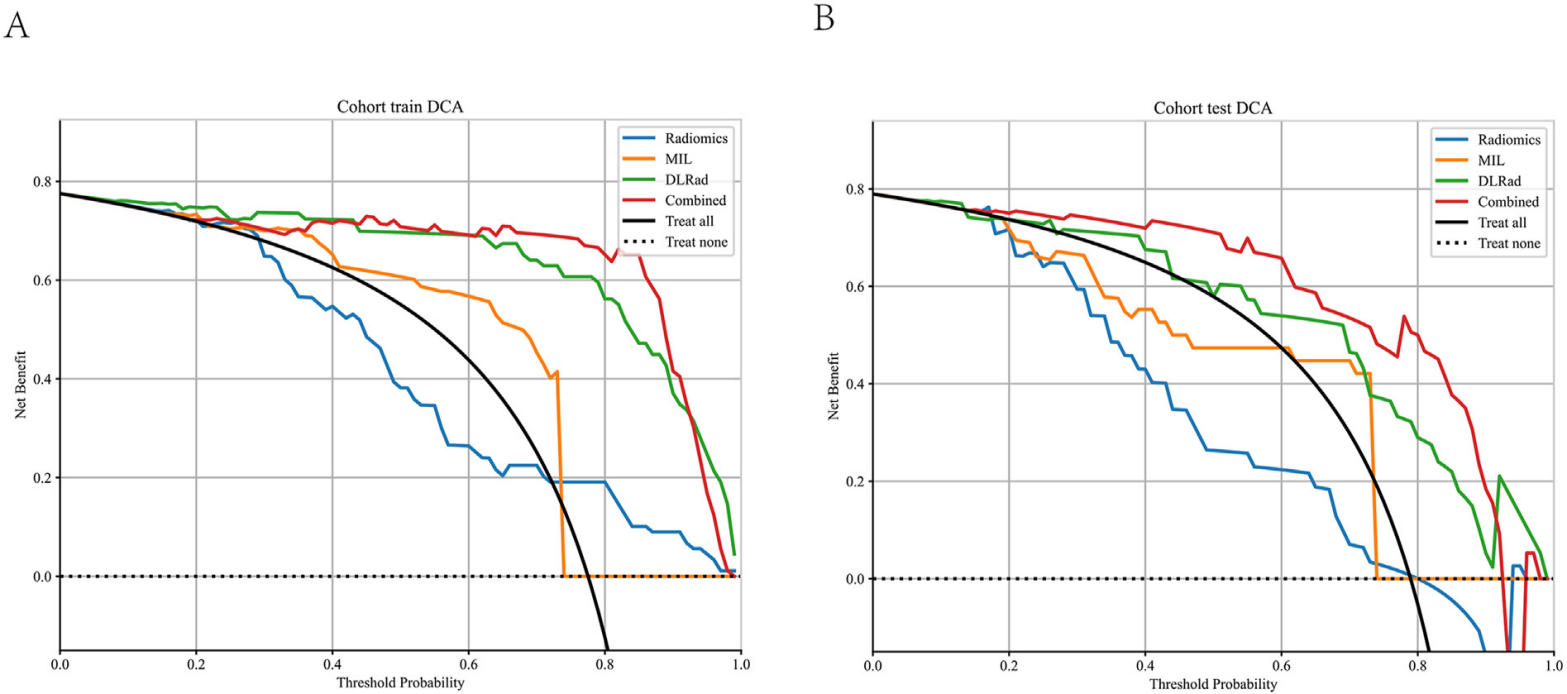
Different signatures' decision curve on test cohort. A. training cohort; B. test cohort.

Fig. 6 presents the Decision Curve Analysis (DCA) for both the training and test sets. The results indicate that our fusion model offers a significant advantage in terms of predictive probabilities. Moreover, compared to other models, it consistently provides a greater potential for net benefit, highlighting its effectiveness.

## Discussion

This study successfully developed a comprehensive predictive model by integrating multi-instance learning (MIL) with radiomics and deep learning techniques to predict outcomes for PD patients undergoing STN-DBS treatment. In our test cohort, the MIL model achieved an AUC of 0.846, outperforming the radiomics model's AUC of 0.825. The DLRad model, which combines MIL and radiomics features, further improved the discriminative ability, achieving an AUC of 0.871. However, when age was added to create the Combined model, the AUC slightly decreased to 0.858. This suggests that while age is a relevant clinical feature, it may not necessarily enhance the predictive accuracy of models that already integrate complex imaging data.

Our study aligns with the latest advancements in medical imaging and machine learning, highlighting the superiority of deep learning models over traditional radiomics. Previous research has shown that while handcrafted features extracted from medical images are useful, they often fail to capture the multi-dimensional nature of the data [20,21]. The integration of multidimensional data and advanced segmentation methodologies plays a crucial role in enhancing the expressive capabilities of models in medical image analysis, particularly in capturing the nuances of complex anatomical structures. Key to this advancement is the use of 2.5D data and the fusion of single T1-weighted imaging, which have been shown to improve the accuracy of predictions and the comprehensiveness of ROI characterization [[22], [23], [24]].

Recent research emphasizes the importance of multi-task learning, indicating that training models across various datasets spanning different organs and imaging modalities can significantly improve a model's ability to calibrate confidence in its predictions [25]. This approach not only enhances the segmentation capabilities of Convolutional Neural Networks (CNNs) but also highlights the efficacy of integrating diverse data sources to refine the model's understanding of medical images. Moreover, the role of deep learning in multimodal medical imaging, particularly in cancer detection, has been increasingly recognized [26,27]. This work emphasizes addressing the challenges that arise in multimodal imaging analysis, including data heterogeneity (the diversity of patients in terms of clinical characteristics and pathological status) and the complexities involved in effectively capturing and integrating diverse diagnostic information. These studies align with the growing consensus on the importance of employing sophisticated models to navigate the intricacies of multimodal data, facilitating a more accurate and comprehensive analysis of medical images [28,29].

In our study, the integration of 2.5D data and advanced segmentation models enhanced feature representation by capturing the intricate nuances of complex ROI structures. This supports the growing discourse on the importance of multimodal data fusion in improving predictive accuracy.

The M2D CNN model represents a novel approach for classifying task-induced fMRI data, demonstrating the capabilities of multi-channel 2D CNNs in handling complex neural datasets [30]. Despite these advances, such models operate within the confines of 2D data processing and may overlook richer contextual information available in the third dimension. By integrating slice information from multiple perspectives, 2.5D deep learning models emerge as a significant evolution from their 2D predecessors. This approach is particularly beneficial in medical imaging, where incorporating adjacent slices provides a more comprehensive understanding of the anatomical structures under study. The 2.5D model effectively bridges the gap between the simplicity of 2D CNNs and the computational demands of full 3D models, offering a balanced solution that leverages depth information without incurring prohibitive computational costs. A prime example of the 2.5D CNN model is its application in segmenting contrast-enhanced lesions in brain MRI scans [31]. These models successfully address challenges posed by small sample datasets, a common issue in medical imaging due to the high cost and complexity of data acquisition. This advancement not only paves the way for more sophisticated and accurate diagnostic tools but also underscores the evolving landscape of deep learning technologies toward more integrated and context-aware models.

The strengths of our study lie in several key areas. Firstly, the use of a relatively large dataset enhances the robustness of our findings. Secondly, the integration of advanced machine learning techniques provides a comprehensive evaluation of model performance. Thirdly, the multimodal feature fusion approach significantly improves the predictive capability compared to traditional single-source data models. Lastly, the rigorous validation processes, including 5-fold cross-validation, SMOTE for sample imbalance, and hyperparameter optimization, ensure the reliability and generalizability of our model.

Our approach involved the use of interpolation techniques to reformat T1-weighted images obtained in the sagittal plane into axial and coronal views. While interpolation does not generate new data, it can enable the model to extract features from different anatomical perspectives. This method is particularly beneficial for improving generalization capability in machine learning models by enhancing training diversity. Some studies have demonstrated similar benefits in medical imaging, suggesting that interpolation can aid in better feature recognition and model robustness [32,33]. This finding could serve as a useful methodology for future work aiming to leverage limited imaging data effectively. In additional, in our slice selection process, we observed that excluding the third slice from the central plane enhanced the model's accuracy. Although, theoretically, this slice should not be excluded outright, our comparative analyses suggested that its omission reduced redundant information and potential noise, factors which could otherwise obscure meaningful data. This finding underscores the importance of careful slice selection and its impact on feature clarity and model accuracy. It highlights a strategic approach in model development that can be explored further in future studies.

Despite its strengths, the study has several limitations. The retrospective nature of data collection may introduce selection biases and limits the causal inference of the observed relationships. Moreover, the generalizability of our findings to other populations or settings remains to be validated through prospective studies. Additionally, potential constraints related to imaging techniques or feature extraction methods might affect the reproducibility of results in different clinical environments [34]. In our study, although data from the Levodopa Challenge Test (LCT) was collected, we initially did not perform a direct comparison between this test and the predictive capabilities of our model. However, subsequent analysis revealed that the model based on LCT yielded an area under the ROC curve (AUC) of only 0.575 (95 ​% CI: 0.473–0.671) (Supplementary 6A), significantly lower than the AUC of our constructed radiomics model. This result suggests that while the LCT is widely recognized as the gold standard for assessing the efficacy of STN-DBS treatment, its predictive capability, especially regarding improvements that fall below conventional clinical significance thresholds, may be influenced by the criteria employed in this study. Further investigation into its limitations and applicability with different cutoff criteria is warranted. Our model demonstrated higher predictive accuracy, suggesting that multimodal data fusion and deep learning techniques may hold greater potential for improving prediction outcomes. This finding underscores the importance of utilizing imaging data to predict STN-DBS results, indicating that radiomics may provide a more effective tool for personalized treatment planning for patients.

In the future, prospective validation studies are crucial to establish the real-world applicability of our predictive models. Further refinement of the models with additional clinical and genetic data could provide deeper insights into PD progression and treatment response. Exploring the potential of similar multimodal approaches in other neurodegenerative disorders could expand the scope of personalized medicine. Additionally, investigating more advanced algorithms and their integration with existing models could provide even more accurate predictions. In this study, patients were categorized based on any improvement in UPDRS-III scores (>0 point increase from baseline) to capture a wide spectrum of post-operative responses and explore all potential predictive factors. Although this approach diverges from conventional clinically significant thresholds, it provides a starting framework to delve into even minimal symptomatic changes, hypothesizing that understanding these could yield broader insights into patient variability. Future investigations could adjust these criteria to focus on clinically meaningful changes, enhancing the clinical robustness of outcome predictions.

For detailed methodologies and additional insights, refer to the supplementary materials provided. Future work could incorporate external validation cohorts to further substantiate the findings and expand the model's applicability.

This study underscores the significant potential of integrating MIL, radiomics, and deep learning to predict STN-DBS outcomes in PD patients. The fusion of multimodal data provides a robust and generalizable predictive model, offering promising avenues for future research and clinical application. By improving the precision and reliability of outcome predictions, our work supports better individualized treatment planning and enhances patient care, paving the way for broader adoption of advanced machine learning techniques in medical diagnostics.

## Author contributions

BWC and ZG jointly completed the experiment and the writing; JMM, ZYW and PC assisted in the writing and followed up patients. CSN YGJ took the overall control of the whole study. All authors contributed to the article and approved the submitted version.

## Data availability

Raw data have been presented in the Supplementary material. Please contact the corresponding author to share the original image data after explaining the purpose.

## Ethics statement

The study was reviewed and approved by Ethics Committee of The First Affiliated Hospital of USTC (2022-RE-154).

## Funding

This paper is supported by the Joint Fund for Medical Artificial Intelligence (No.: MAI2023Q023), Excellent Scientific research and innovation Team Project in 10.13039/100015791Anhui Province (2023AH010080).

## Declaration of competing interest

The authors declare that they have no known competing financial interests or personal relationships that could have appeared to influence the work reported in this paper.
