# Supplementary material for: Application of multimodal deep learning and multi-instance learning fusion techniques in predicting STN-DBS outcomes for Parkinson's disease patients
Source: Neurotherapeutics. 2024 Oct 16;21(6):e00471. doi: 10.1016/j.neurot.2024.e00471 (PMC11585874; doi:10.1016/j.neurot.2024.e00471)

## Supplementary

### 2A. Results of MIL Signature

Table1. Slice level results of different CNN models.

| ModelName | Acc | AUC | 95% CI | Sensitivity | Specificity | PPV | NPV | Cohort |
| --- | --- | --- | --- | --- | --- | --- | --- | --- |
| resnet101 | 0.529 | 0.623 | 0.5811-0.6650 | 0.468 | 0.740 | 0.861 | 0.287 | train |
| resnet101 | 0.424 | 0.533 | 0.4631-0.6029 | 0.320 | 0.812 | 0.865 | 0.242 | test |
| densenet121 | 0.681 | 0.666 | 0.6251-0.7073 | 0.725 | 0.530 | 0.842 | 0.358 | train |
| densenet121 | 0.639 | 0.607 | 0.5392-0.6744 | 0.687 | 0.462 | 0.827 | 0.282 | test |
| inception_v3 | 0.493 | 0.604 | 0.5600-0.6490 | 0.412 | 0.775 | 0.863 | 0.276 | train |
| inception_v3 | 0.742 | 0.552 | 0.4718-0.6332 | 0.850 | 0.337 | 0.828 | 0.375 | test |

Fig1. ROC of different models in slice level prediction.


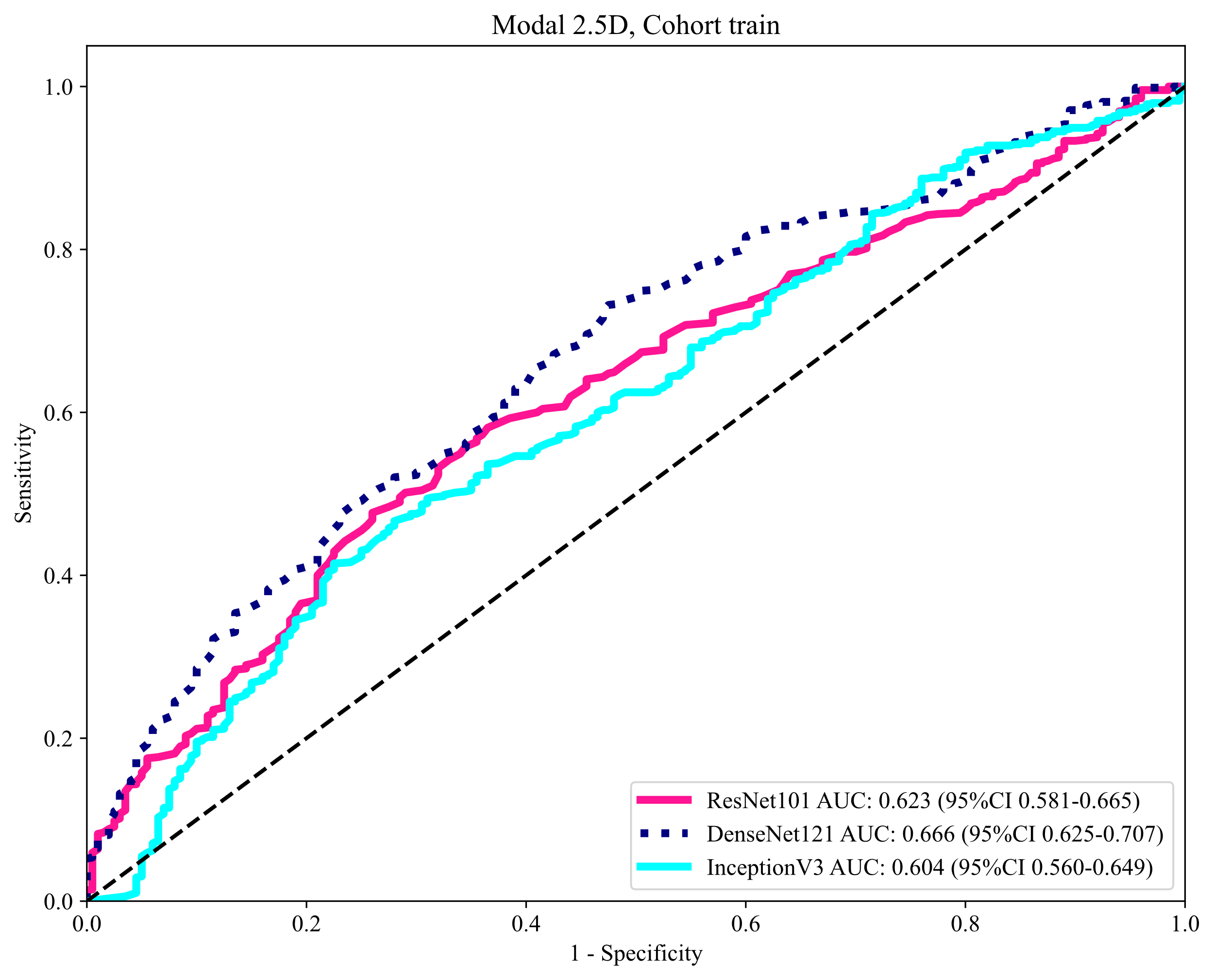

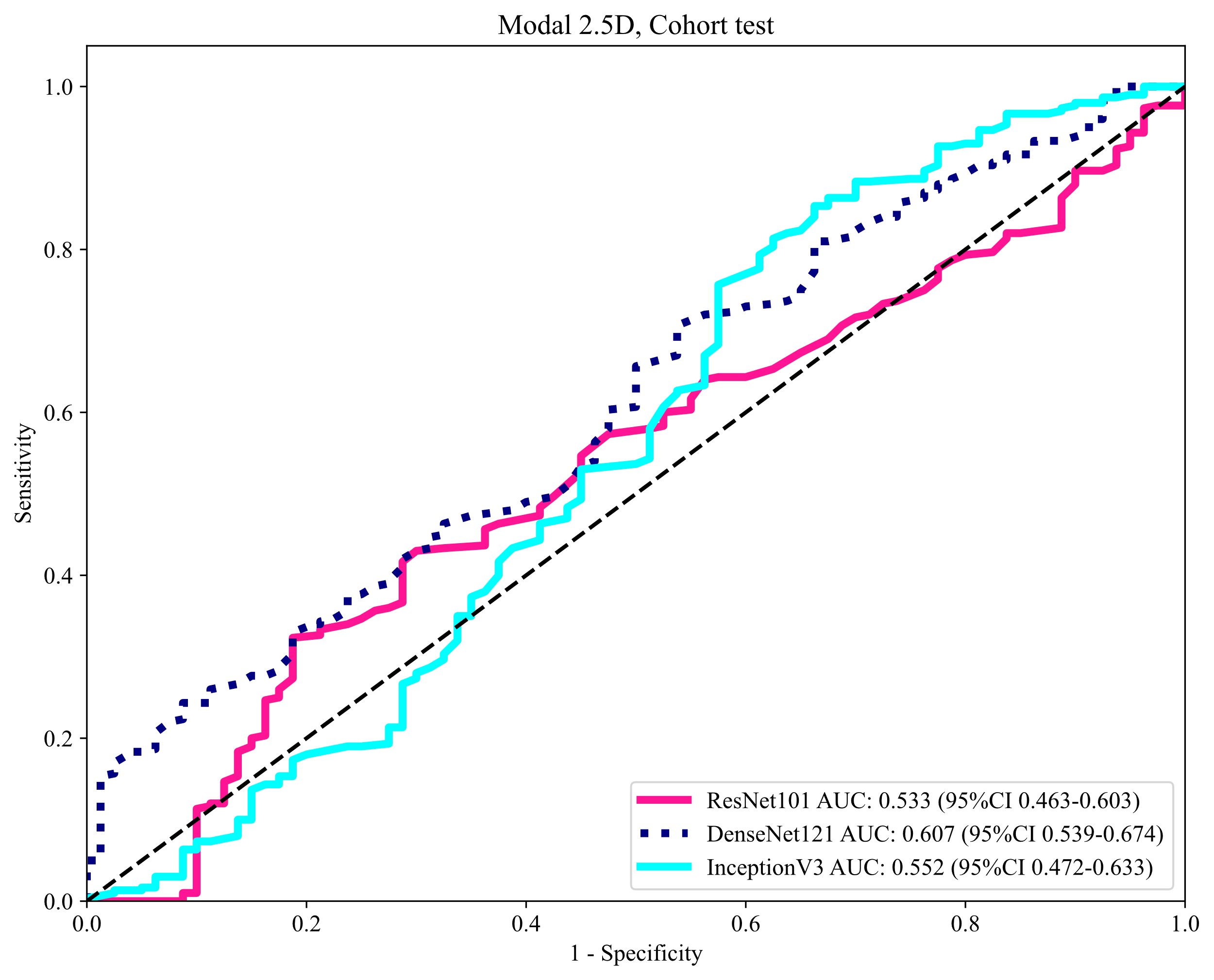


#### Grad-CAM

Figure 2. displays Grad-CAM visualizations for two representative samples, demonstrating how the model selectively focuses on different regions of the images to make its predictions. This visualization is crucial for understanding the model's attention mechanism in practical applications.


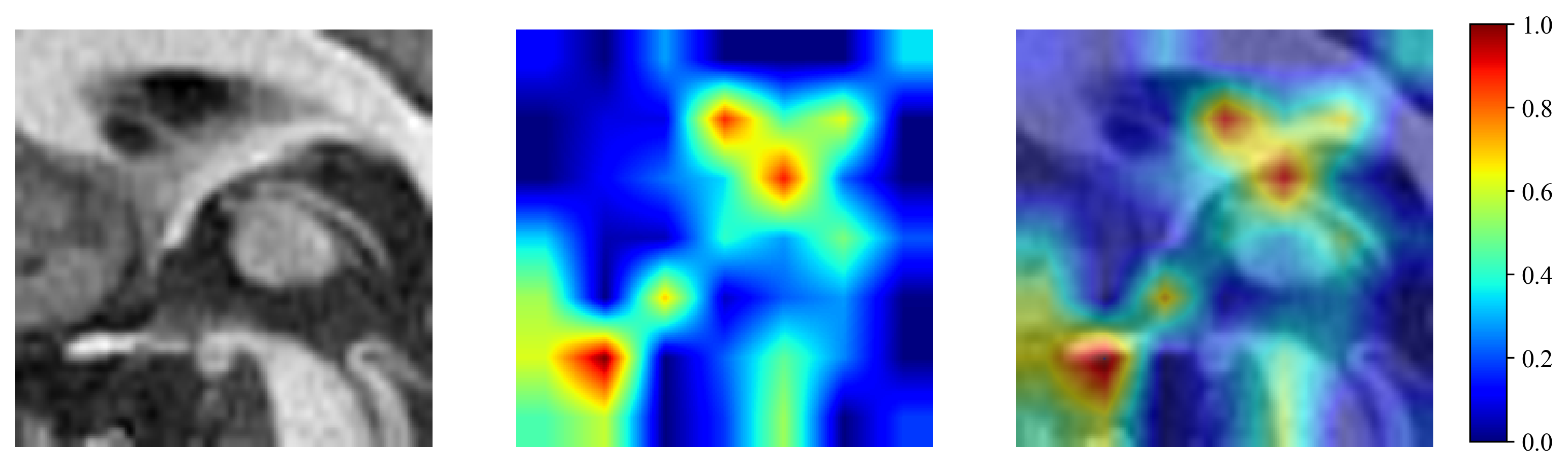


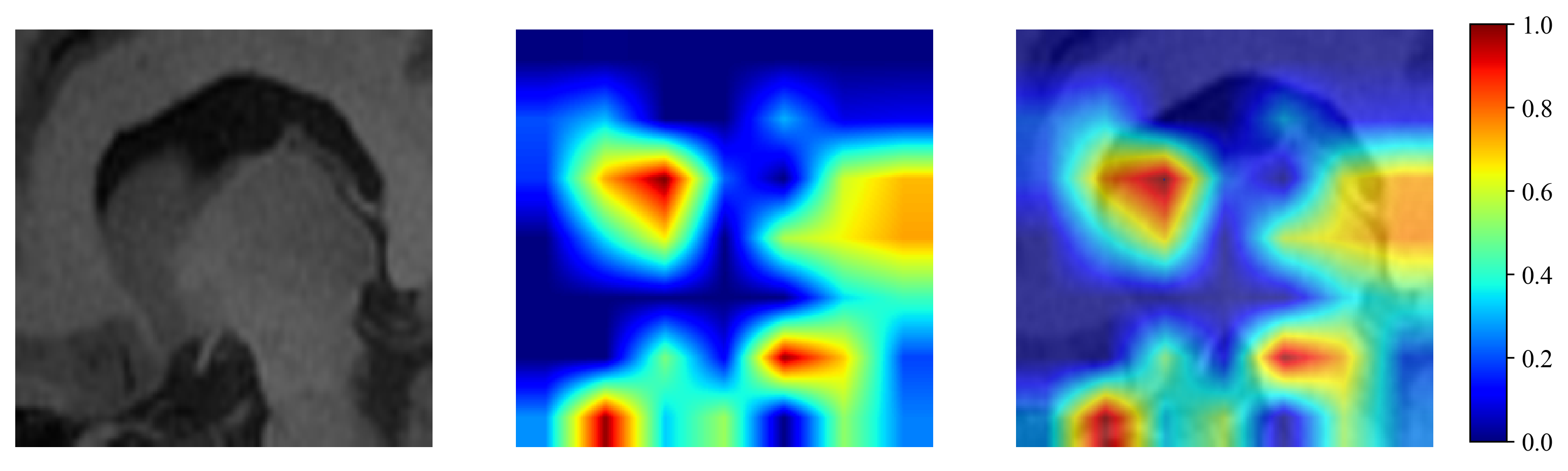


#### MIL Fusion Results

Table3 metrics of different machine learning method in MIL models

| model_name | Accuracy | AUC | 95% CI | Sensitivity | Specificity | PPV | NPV | Cohort |
| --- | --- | --- | --- | --- | --- | --- | --- | --- |
| LR | 0.798 | 0.826 | 0.713 - 0.939 | 0.826 | 0.700 | 0.905 | 0.538 | train |
| LR | 0.684 | 0.767 | 0.614 - 0.920 | 0.633 | 0.875 | 0.950 | 0.389 | test |
| SVM | 0.831 | 0.847 | 0.736 - 0.957 | 0.870 | 0.700 | 0.909 | 0.609 | train |
| SVM | 0.684 | 0.846 | 0.721 - 0.971 | 0.600 | 1.000 | 1.000 | 0.400 | test |
| RandomForest | 0.876 | 0.878 | 0.770 - 0.986 | 0.913 | 0.750 | 0.926 | 0.714 | train |
| RandomForest | 0.737 | 0.817 | 0.666 - 0.967 | 0.800 | 0.500 | 0.857 | 0.400 | test |

Fig3. Cross Validation results for parameter gird search, ROC of different models in patient level prediction.


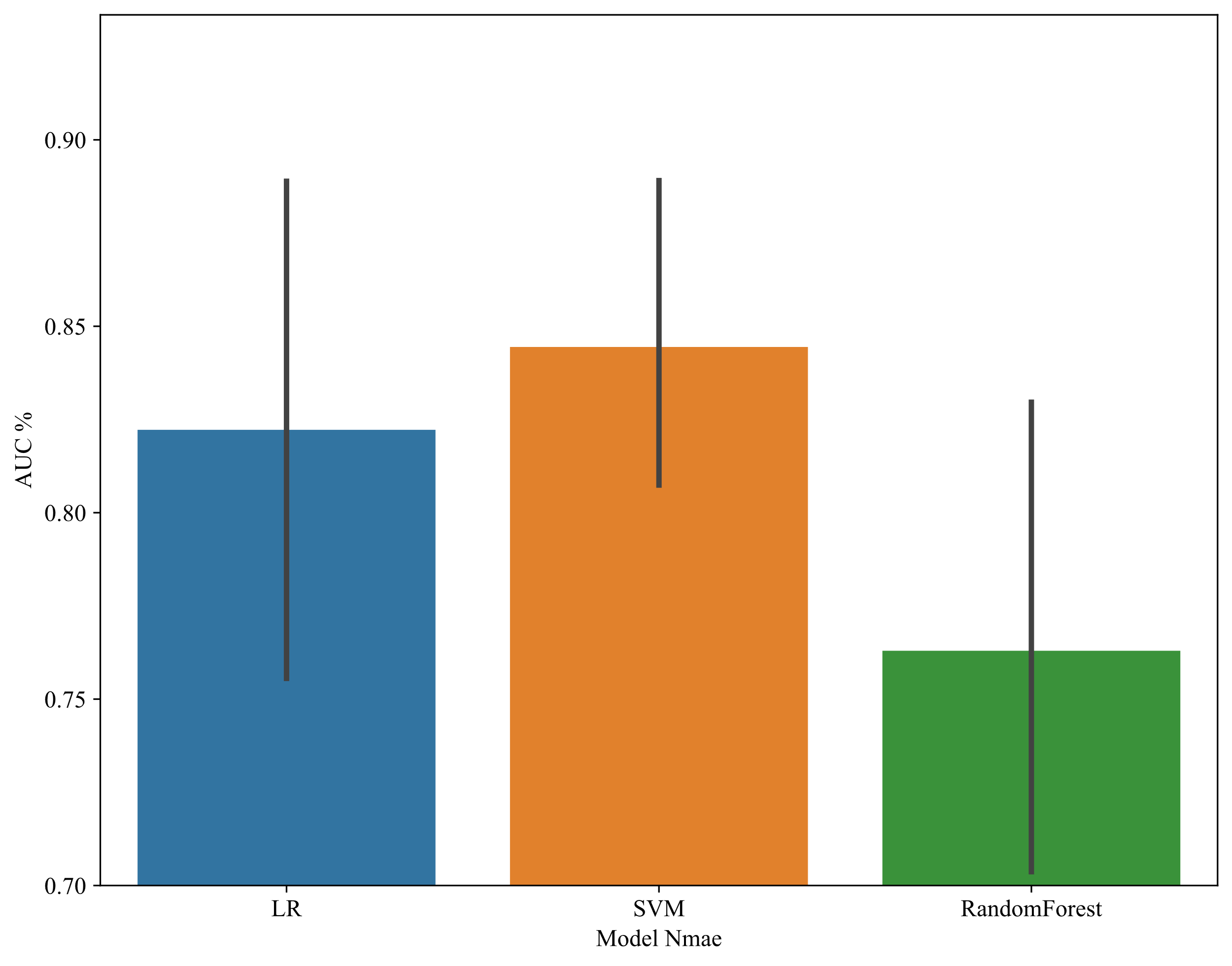

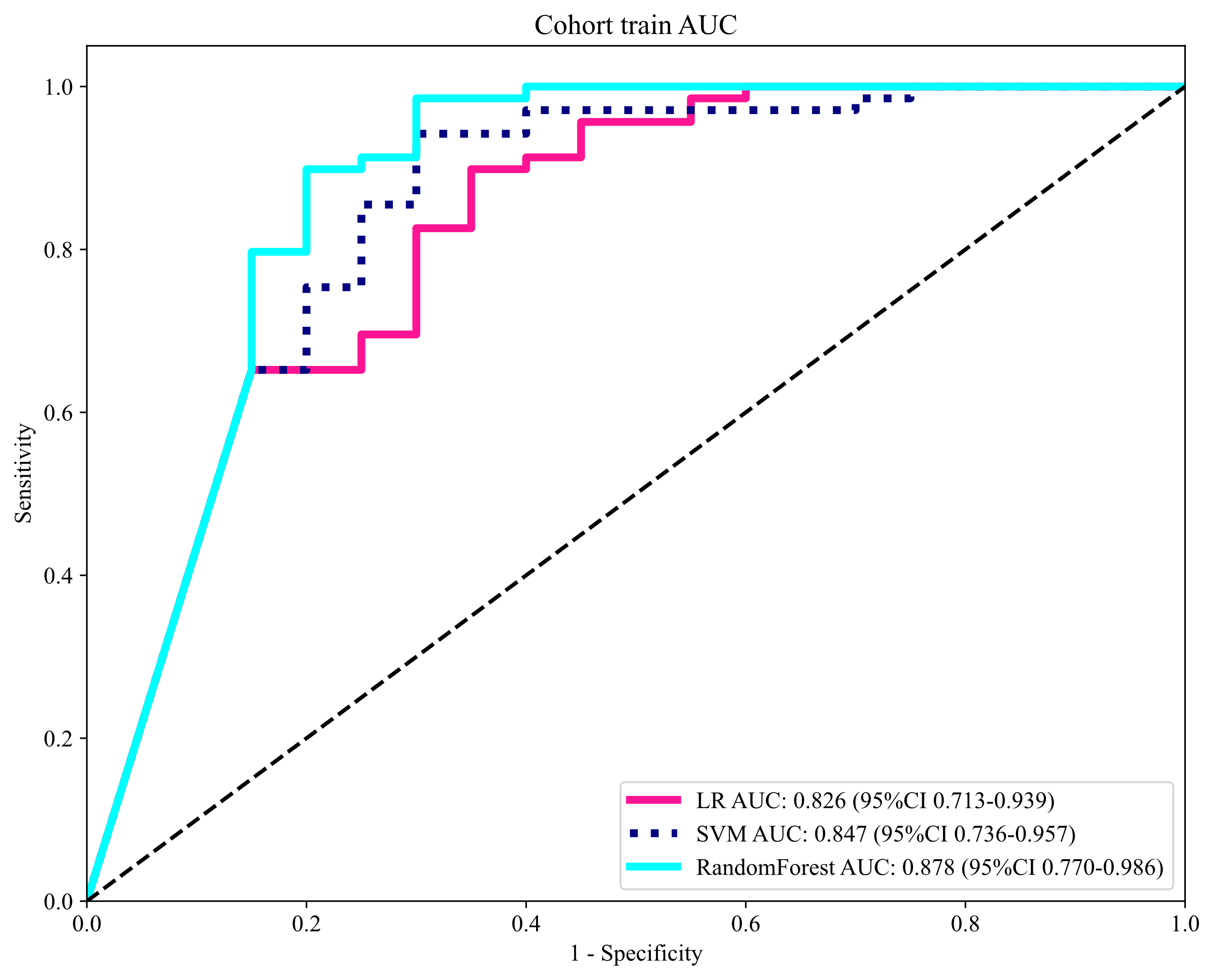

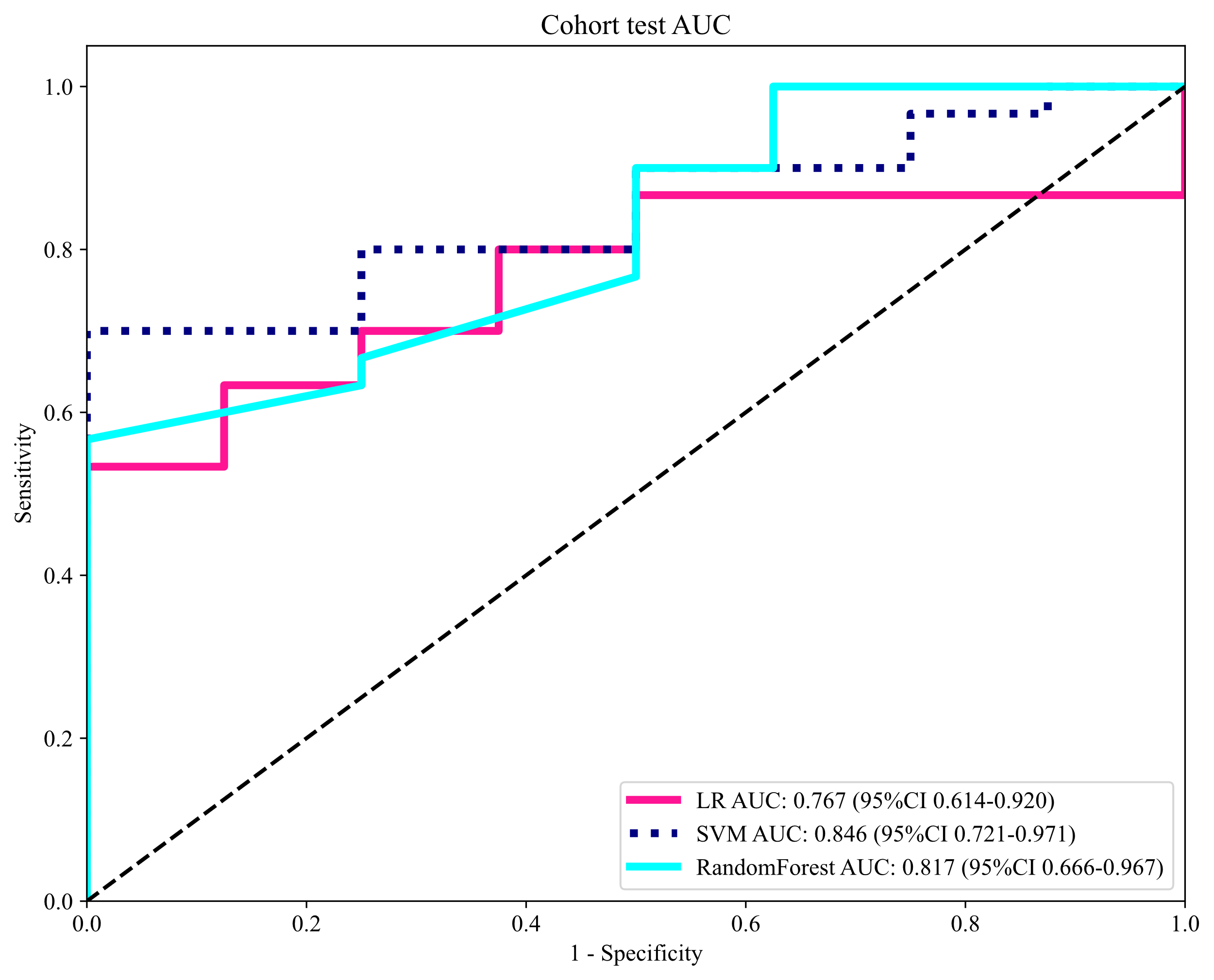


### 2B. Comparison of Different Fusion Method

| Fusion Method | Acc | AUC | 95% CI | Sensitivity | Specificity | PPV | NPV | Cohort |
| --- | --- | --- | --- | --- | --- | --- | --- | --- |
| MIL | 0.831 | 0.847 | 0.7362-0.9573 | 0.870 | 0.700 | 0.909 | 0.609 | train |
| mean | 0.775 | 0.770 | 0.6667-0.8724 | 1.000 | 0.000 | 0.775 | 0.000 | train |
| max | 0.775 | 0.696 | 0.5773-0.8155 | 1.000 | 0.000 | 0.775 | 0.000 | train |
| min | 0.708 | 0.693 | 0.5737-0.8132 | 0.797 | 0.400 | 0.821 | 0.364 | train |
| MIL | 0.684 | 0.846 | 0.7210-0.9707 | 0.600 | 1.000 | 1.000 | 0.400 | test |
| mean | 0.632 | 0.679 | 0.4590-0.8994 | 0.633 | 0.625 | 0.864 | 0.312 | test |
| max | 0.789 | 0.583 | 0.3667-0.8000 | 1.000 | 0.000 | 0.789 | 0.000 | test |
| min | 0.237 | 0.573 | 0.3275-0.8184 | 0.033 | 1.000 | 1.000 | 0.216 | test |


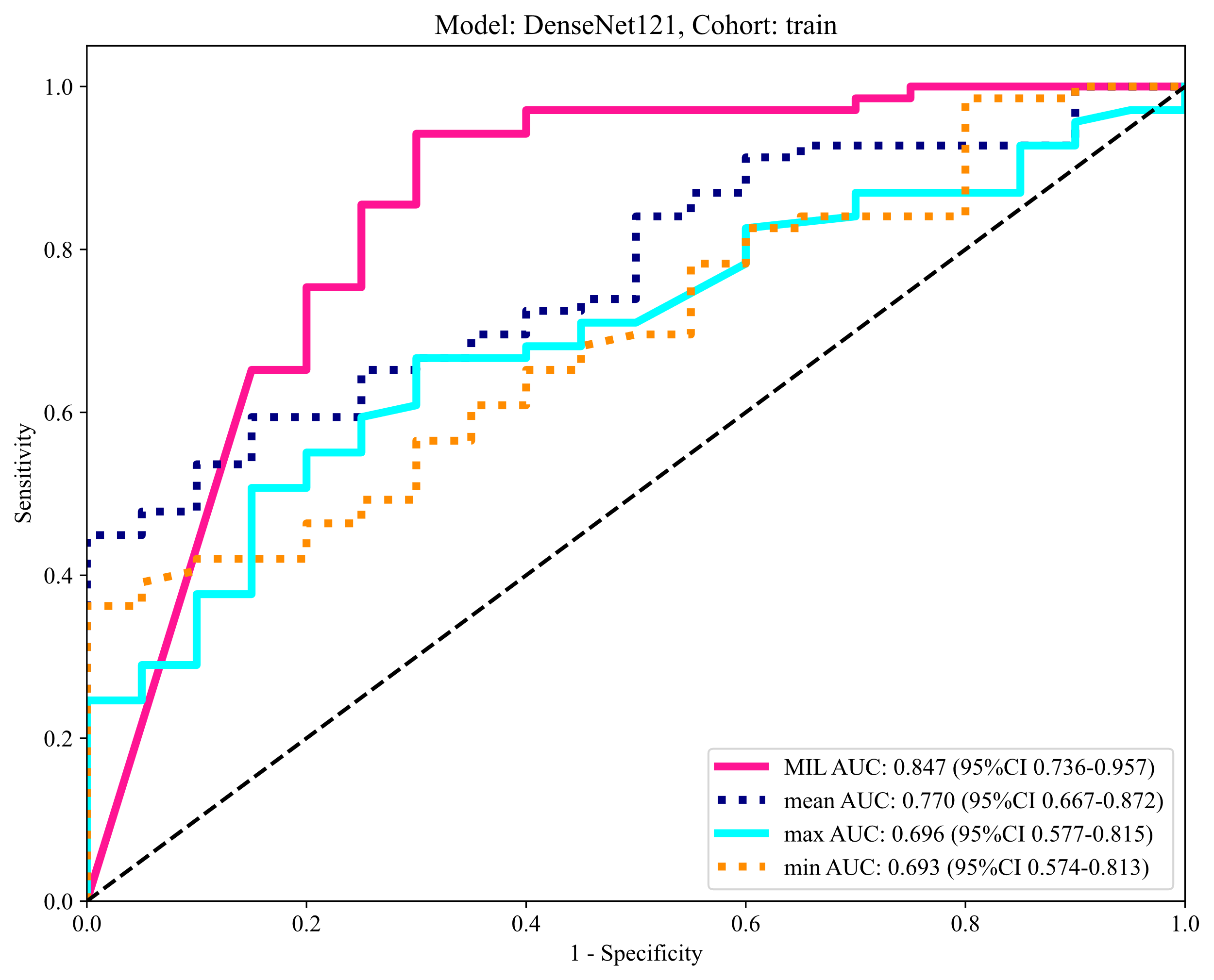

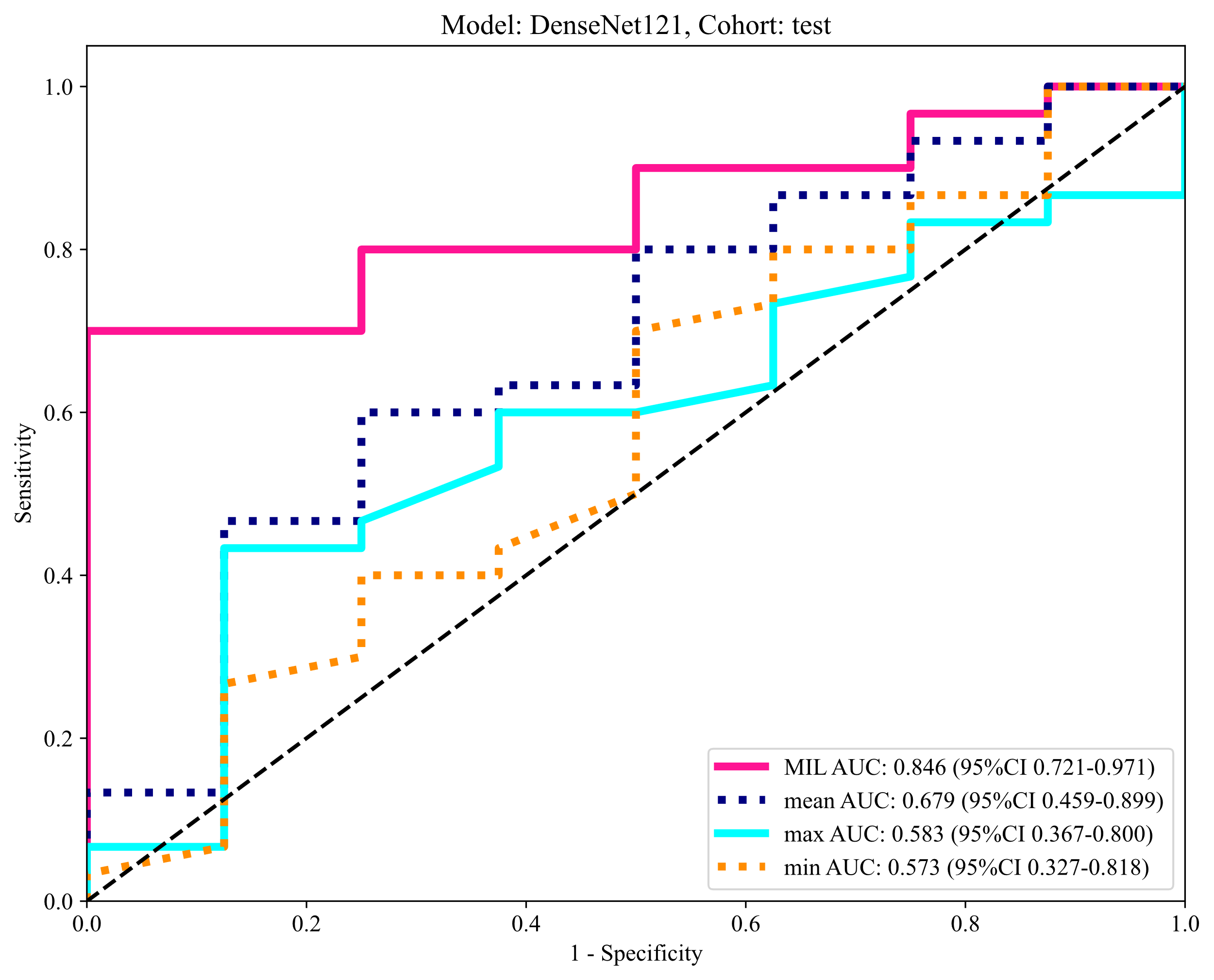


### 3A. Building Radiomics Model

Table1 metrics of different machine learning method in Radiomics models

| model_name | Accuracy | AUC | 95% CI | Sensitivity | Specificity | PPV | NPV | Cohort |
| --- | --- | --- | --- | --- | --- | --- | --- | --- |
| LR | 0.798 | 0.826 | 0.713 - 0.939 | 0.826 | 0.700 | 0.905 | 0.538 | train |
| LR | 0.684 | 0.767 | 0.614 - 0.920 | 0.633 | 0.875 | 0.950 | 0.389 | test |
| SVM | 0.831 | 0.847 | 0.736 - 0.957 | 0.870 | 0.700 | 0.909 | 0.609 | train |
| SVM | 0.684 | 0.846 | 0.721 - 0.971 | 0.600 | 1.000 | 1.000 | 0.400 | test |
| RandomForest | 0.876 | 0.878 | 0.770 - 0.986 | 0.913 | 0.750 | 0.926 | 0.714 | train |
| RandomForest | 0.737 | 0.817 | 0.666 - 0.967 | 0.800 | 0.500 | 0.857 | 0.400 | test |

Fig2. ROC of different models radiomics signature.


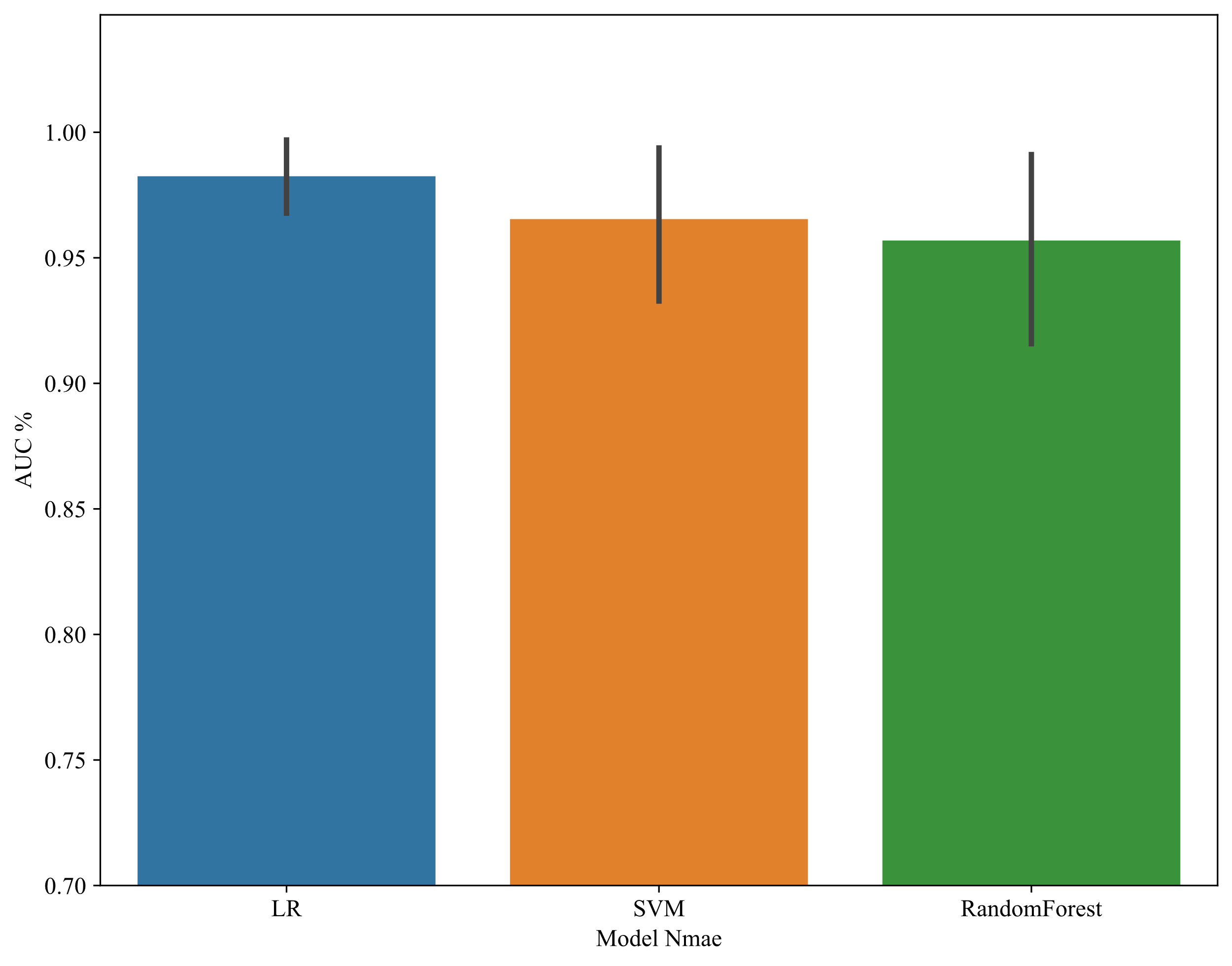

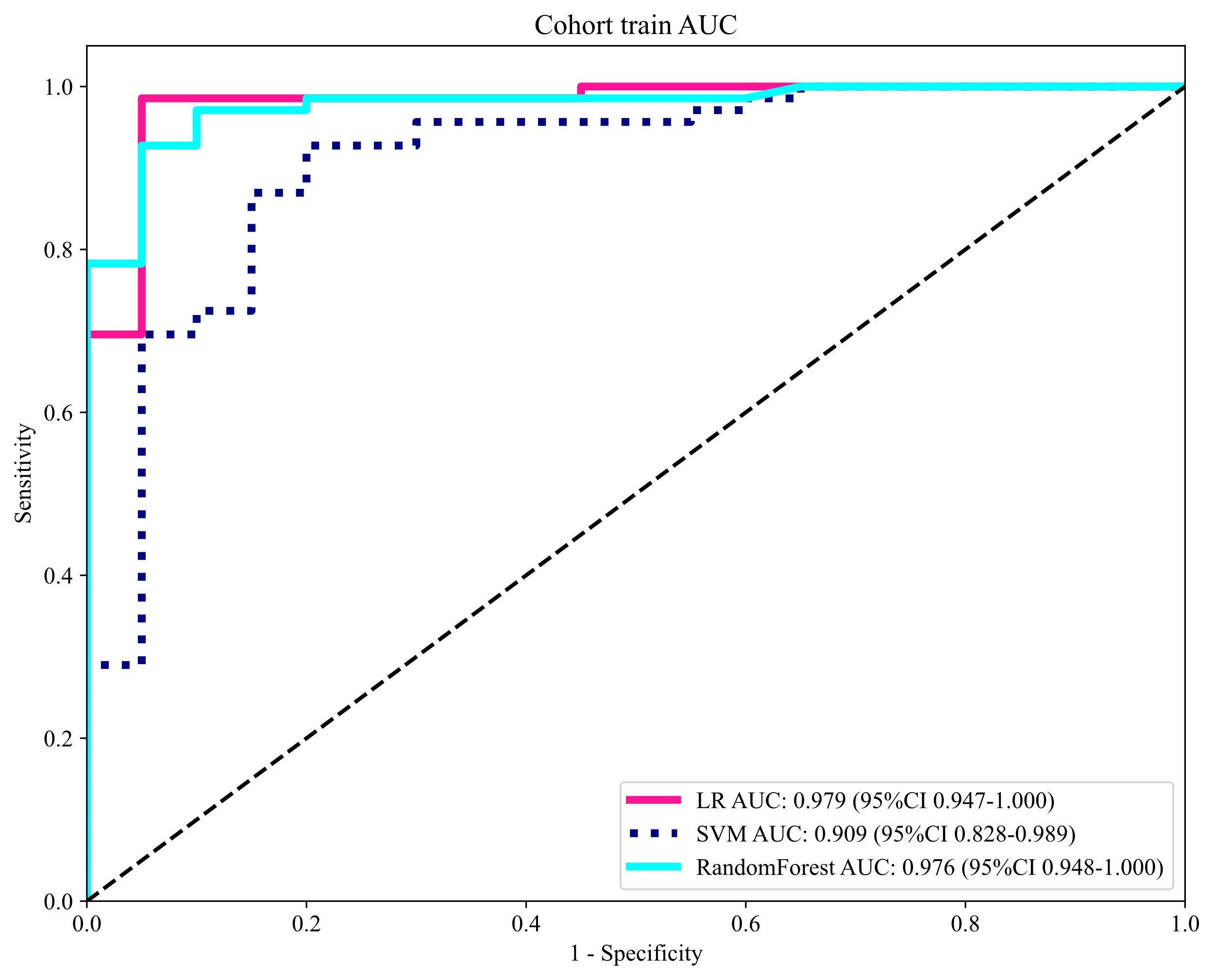

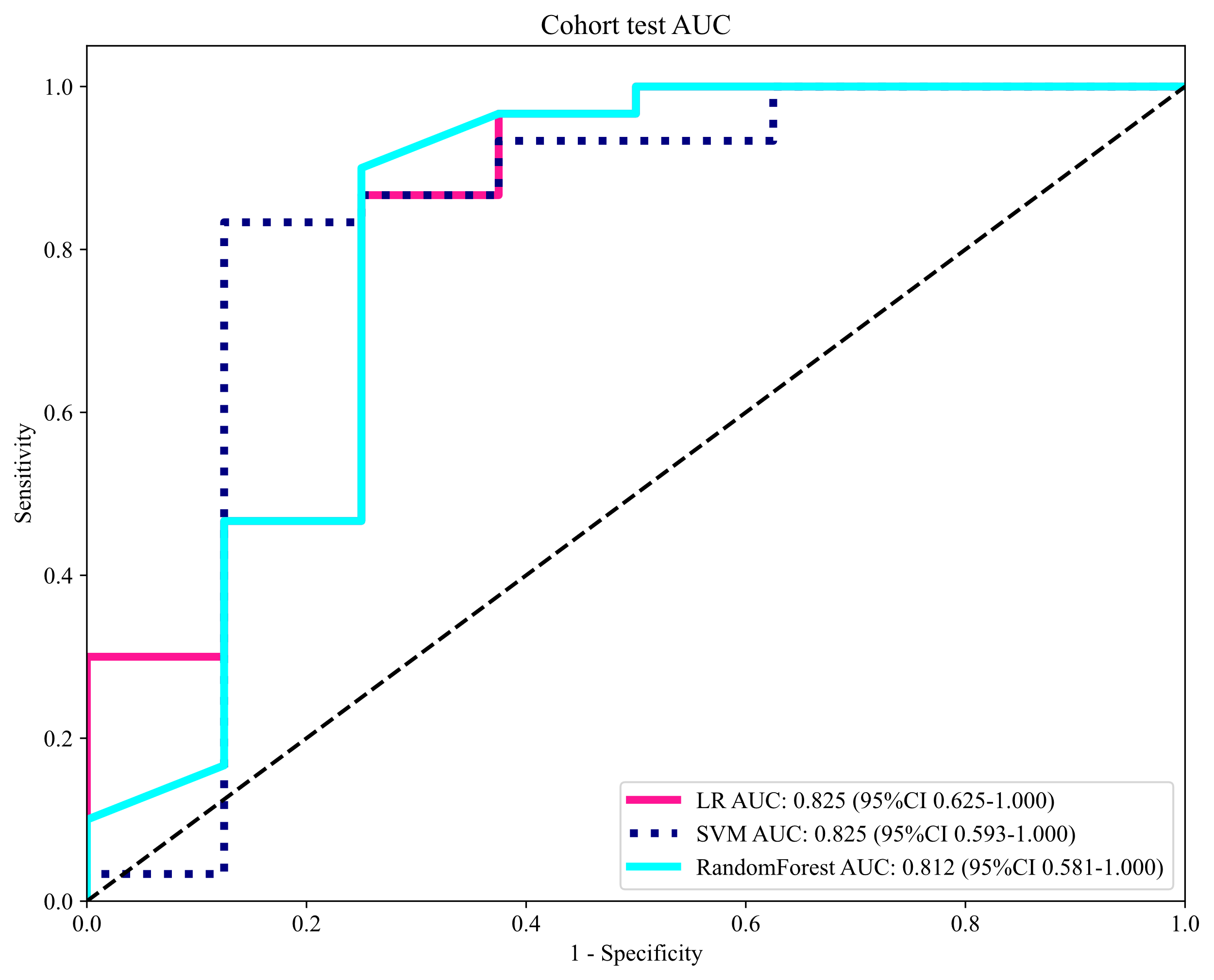


### 4A. Details of Deep Learning Radiomics Signature

Table1 metrics of different machine learning method in Radiomics models

| model_name | Accuracy | AUC | 95% CI | Sensitivity | Specificity | PPV | NPV | Cohort |
| --- | --- | --- | --- | --- | --- | --- | --- | --- |
| LR | 0.809 | 0.986 | 0.961 - 1.000 | 0.754 | 1.000 | 1.000 | 0.541 | train |
| LR | 0.632 | 0.854 | 0.687 - 1.000 | 0.600 | 0.750 | 0.900 | 0.333 | test |
| SVM | 0.921 | 0.983 | 0.964 - 1.000 | 0.913 | 0.950 | 0.984 | 0.760 | train |
| SVM | 0.789 | 0.871 | 0.698 - 1.000 | 0.800 | 0.750 | 0.923 | 0.500 | test |
| RandomForest | 0.966 | 0.992 | 0.979 - 1.000 | 0.971 | 0.950 | 0.985 | 0.905 | train |
| RandomForest | 0.711 | 0.812 | 0.672 - 0.953 | 0.800 | 0.375 | 0.828 | 0.333 | test |

Fig2. ROC of different models DLRad signature.


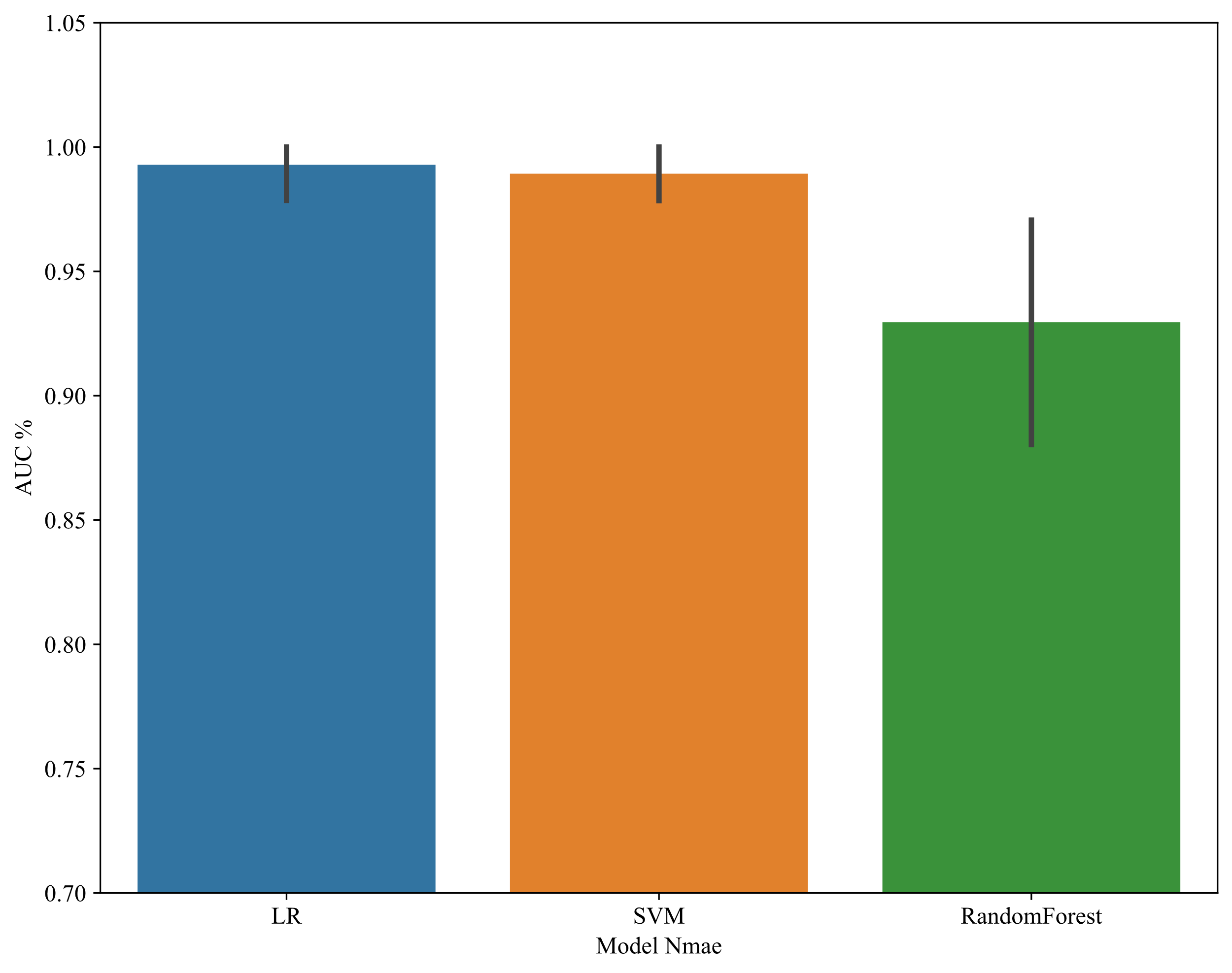

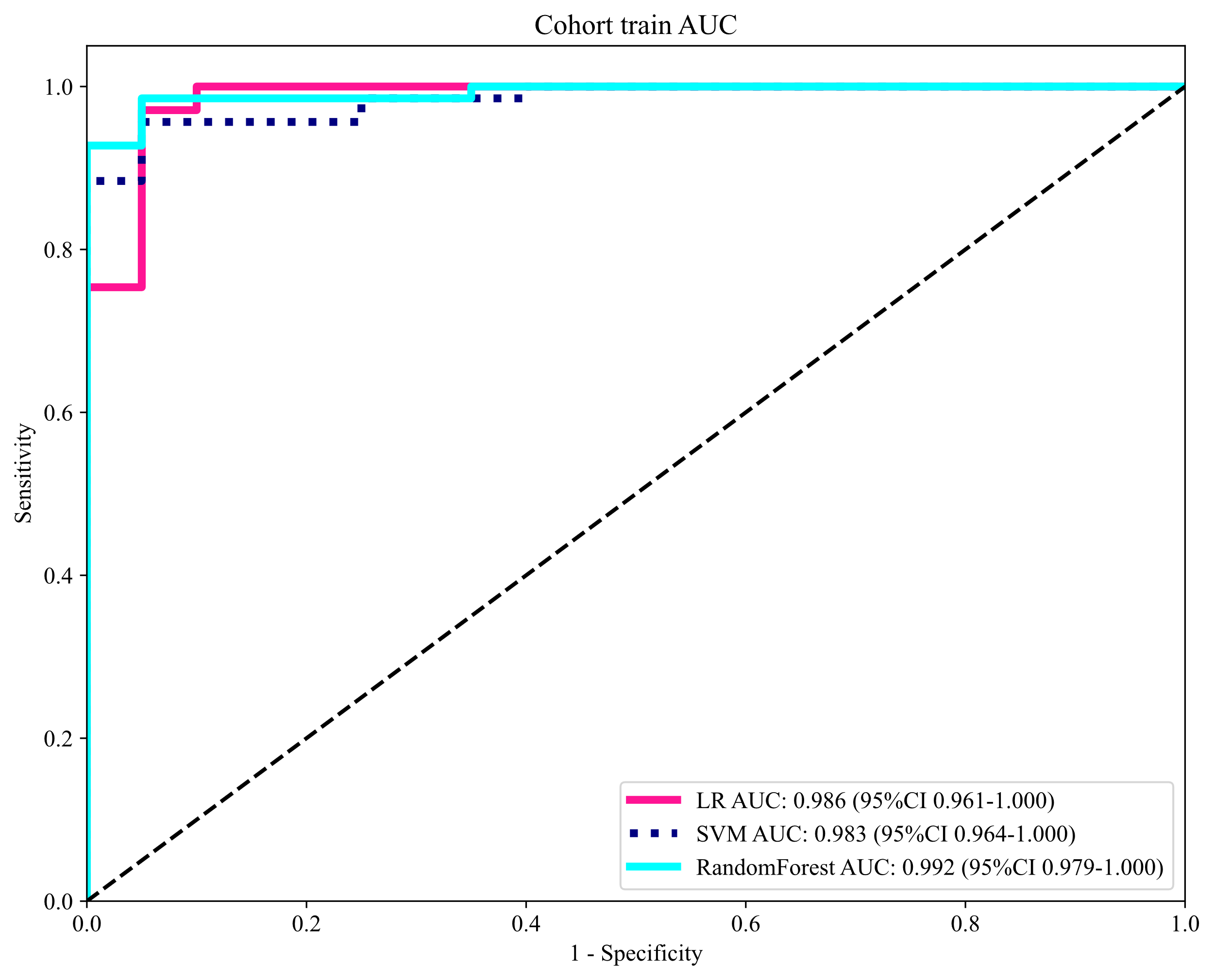

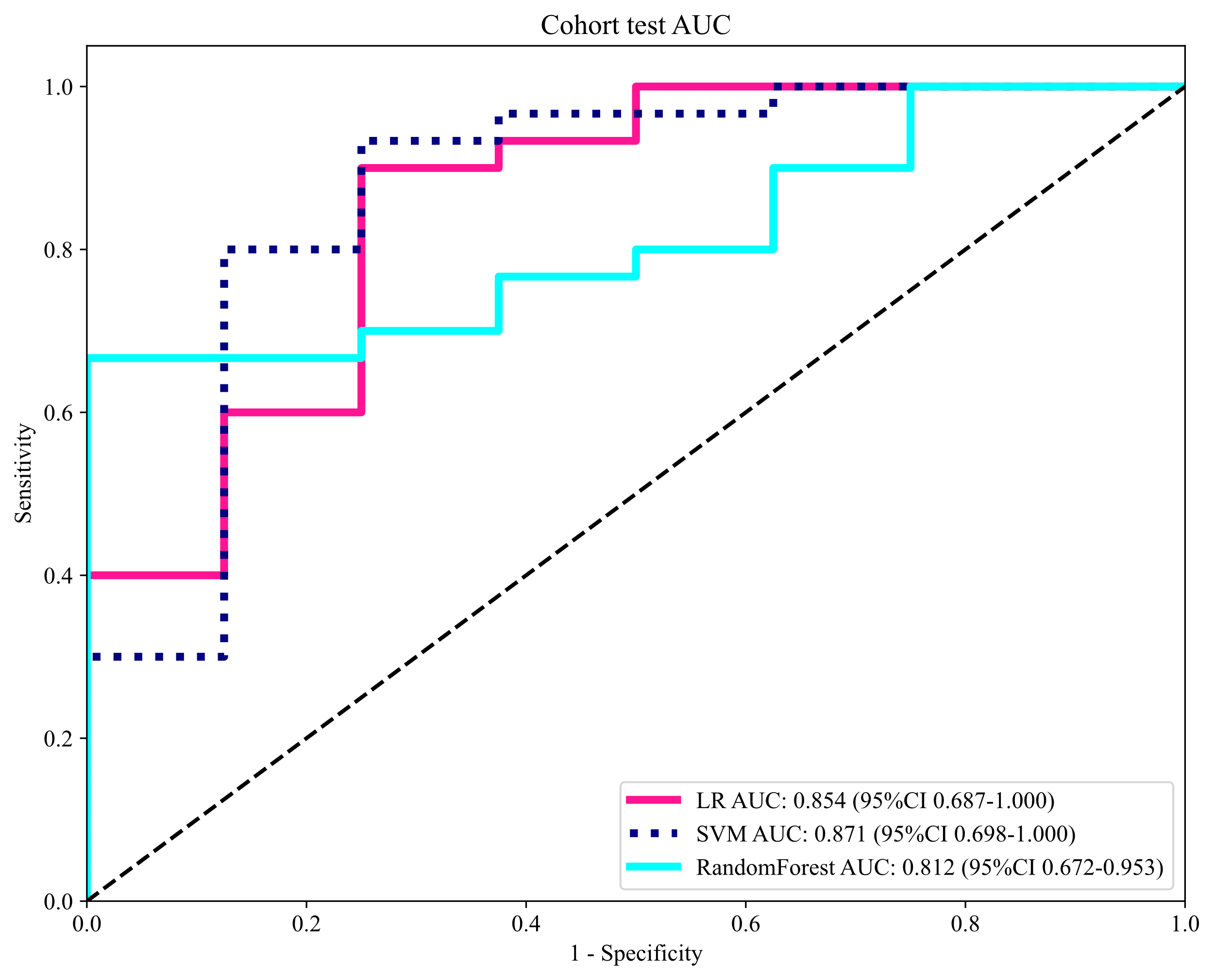


### 5A. The confusion matrices for both the training and testing phases (A. Rad; B.DLRad; C. MIL )


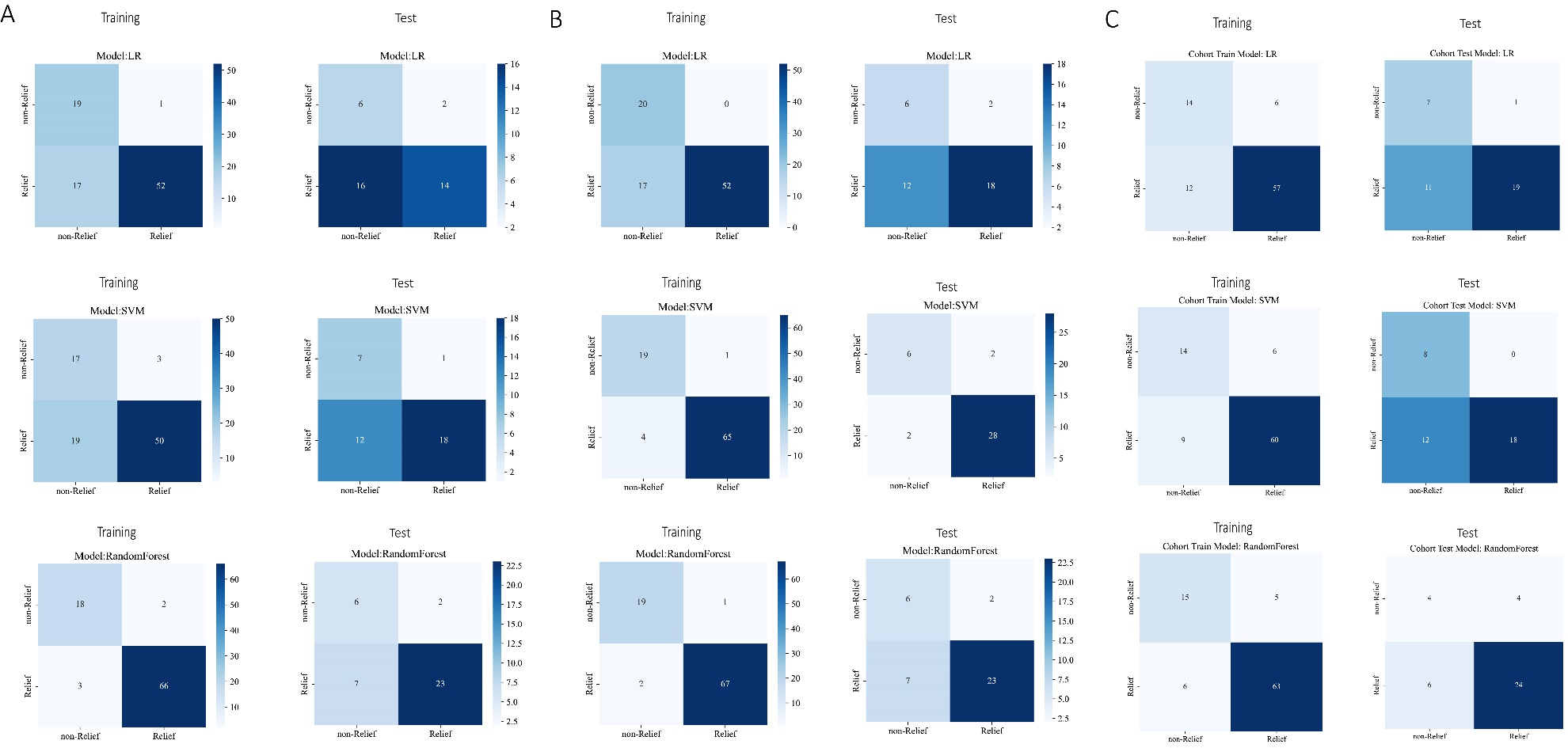


### 6A. The model based on LCT yielded an area under the ROC curve
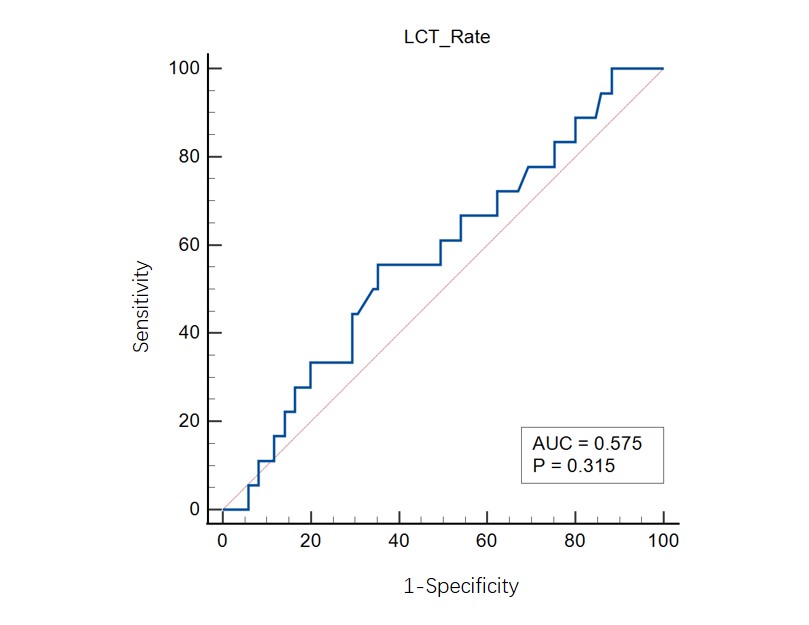

Supplement: Multimedia component 1 [file mmc1.docx]
